# Deleterious variation shapes the genomic landscape of introgression

**DOI:** 10.1371/journal.pgen.1007741

**Published:** 2018-10-22

**Authors:** Bernard Y. Kim, Christian D. Huber, Kirk E. Lohmueller

**Affiliations:** 1 Department of Ecology and Evolutionary Biology, University of California, Los Angeles, California, United States of America; 2 Interdepartmental Program in Bioinformatics, University of California, Los Angeles, California, United States of America; 3 Department of Human Genetics, David Geffen School of Medicine, University of California, Los Angeles, California, United States of America; Aarhus University, DENMARK

## Abstract

While it is appreciated that population size changes can impact patterns of deleterious variation in natural populations, less attention has been paid to how gene flow affects and is affected by the dynamics of deleterious variation. Here we use population genetic simulations to examine how gene flow impacts deleterious variation under a variety of demographic scenarios, mating systems, dominance coefficients, and recombination rates. Our results show that admixture between populations can temporarily reduce the genetic load of smaller populations and cause increases in the frequency of introgressed ancestry, especially if deleterious mutations are recessive. Additionally, when fitness effects of new mutations are recessive, between-population differences in the sites at which deleterious variants exist creates heterosis in hybrid individuals. Together, these factors lead to an increase in introgressed ancestry, particularly when recombination rates are low. Under certain scenarios, introgressed ancestry can increase from an initial frequency of 5% to 30–75% and fix at many loci, even in the absence of beneficial mutations. Further, deleterious variation and admixture can generate correlations between the frequency of introgressed ancestry and recombination rate or exon density, even in the absence of other types of selection. The direction of these correlations is determined by the specific demography and whether mutations are additive or recessive. Therefore, it is essential that null models of admixture include both demography and deleterious variation before invoking other mechanisms to explain unusual patterns of genetic variation.

## Author summary

Individuals from distinct populations sometimes will produce fertile offspring and will exchange genetic material in a process called hybridization. Genomes of hybrid individuals often show non-random patterns of hybrid ancestry across the genome, where some regions have a high frequency of ancestry from the second population and other regions have less. Typically, this pattern has been attributed to adaptive introgression, where beneficial genetic variants are passed from one population to the other, or to genomic incompatibilities between these distinct species. However, other mechanisms could lead to these heterogeneous patterns of ancestry in hybrids. Here we use simulations to investigate whether deleterious mutations affect the patterns of introgressed ancestry across genomes. We show that when ancestry from a larger population is added to a smaller population, the ancestry from the larger population dramatically increases in frequency because it carries fewer deleterious mutations. This occurs even in the absence of beneficial mutations in either population. Additionally, we show that differences in sex chromosome evolution relative to autosomes, or differences in mating system, can affect patterns of introgression in similar ways. Our study argues that deleterious mutations should be included in population genetic models used to identify unusual regions of the genome that appear to be under selection in hybrids.

## Introduction

There is tremendous interest in quantifying the effects that demographic history has had on the patterns and dynamics of deleterious variation and genetic load [1–8]. Several studies have suggested that recent human demography has had little impact on load [9,10] while others have suggested weak, but subtle, differences between human populations [11–15]. All of these studies have typically focused on how population size changes, such as expansions and bottlenecks, have affected deleterious variation. Other types of complex demography, however, have received considerably less attention.

In particular, gene flow may be important for shaping patterns of deleterious variation. Population admixture, or hybridization between closely related species, appears to be quite common in nature [16] and has had a significant role in shaping human genomes [17]. Gene flow alone can subtly change the effects of selection on deleterious variation [13], but should have notable fitness consequences if deleterious variation is distributed differently between admixing populations. For example, Neanderthals likely had a higher genetic load than coincident human populations due to the former’s smaller long-term population size [18,19]. As a result, it is thought that gene flow from Neanderthals into the ancestors of modern humans could have increased the genetic load of some human populations by 0.5% [18], and that linked selection removed much of Neanderthal ancestry from humans since that time. In contrast, domesticated species likely have increased genetic load due to domestication bottlenecks and hitchhiking of deleterious alleles with artificially selected variants [20–22]. Gene flow from wild populations could alleviate the genetic load of domesticated species, and increases in the frequency of wild-population ancestry should be observed in the domesticated population [23]. Such changes in patterns of introgression are important to consider when studying how natural selection shapes the evolution of hybrid ancestry, a major goal in evolutionary biology.

Differences in the distribution of deleterious variation between hybridizing populations is one reason why natural selection may shape the evolution of hybrid ancestry. Hybridization can also decrease the fitness of a population, for instance, if the parent lineages have diverged significantly and evolved genomic incompatibilities, or if parent lineages have evolved under unique and strong selective pressures in different environments. In both cases, linked selection removes hybrid ancestry especially in regions of low recombination and high functional density [24–26]. This creates genome wide, negative correlations between the local recombination rate, or functional density, and the frequency of introgressed ancestry, a pattern that is observed in humans [24,26,27], swordtail fish [26], and mice [28]. The similar outcomes of both these processes mean that models of selection on deleterious variation should be considered before interpreting genomic patterns of introgression as evidence of divergence and speciation.

Another complication to studying the effects of deleterious mutations on introgression is that strongly deleterious new mutations are more likely to be fully or partially recessive [29–31]. Furthermore, dominance coefficients vary between species. New mutations in humans [14] are more likely to be additive than new mutations with the same selection coefficient in Arabidopsis [31]. If some proportion of deleterious recessive variants is private to a population, admixed populations could experience heterosis when recessive variants are masked (heterozygous) in hybrid individuals [32]. As a result, heterosis may participate in a tug-of-war on hybrid ancestry with additive variants by increasing the frequency of linked ancestry [18], increasing apparent migration rates in regions linked to selected variants [33,34], particularly when gene flow occurs in a highly structured population [35]. Heterosis should also increase the probability that introgressed ancestry will persist in an admixed population, even if the introgressed ancestry contains more deleterious alleles [18]. Given the extent to which hybridization is thought to be common to all species [16], with levels of shared polymorphism in taxa such as *Arabidopsis* motivating arguments for the bifurcating species concept to be revoked [36], it is crucial to understand the contribution of heterosis to patterns of hybrid ancestry.

Hybridization also transfers novel adaptive variants between evolutionarily distinct lineages [37]. In humans, many Neanderthal variants are thought to be adaptive [38], possibly affecting phenotypes such as skin pigmentation [39,40], the response to oxygen levels at high altitudes [41], and immunity to pathogens [42,43]. In this case, the introduction of beneficial alleles via gene flow will also oppose the effect of linked selection from deleterious variation, since introgressed ancestry would increase in frequency by hitchhiking with adaptively introgressed variants. Interestingly, North American populations of *Drosophila melanogaster* exhibit an overall enrichment for introgressed African ancestry in genomic regions of low recombination [44,45]. The divergence time between these two *D*. *melanogaster* populations is small, and so selection on hybrid individuals may be driven by adaptive variants that arose over shorter time scales than genomic incompatibilities. On the other hand, no correlation between recombination rate and introgression is observed in invasive Californian sunflowers [46]. How selection against additive deleterious variation, selection for adaptive variants, and heterosis interact to determine these genomic patterns is unknown.

The objective of this study is to develop a clearer idea for null models of the dynamics of introgression in hybridizing populations while considering the effect of deleterious variants on fitness. Specifically, we aim to understand how selection on introgressed ancestry is determined by differences in the effective population size, mating system, genome structure, recombination rate, distribution of fitness effects, and distribution of dominance coefficients. Previous simulation and empirical work have shown that for at least some systems, deleterious variation is a significant modulator of gene flow [18,19,23,25,26], but few studies have investigated these questions outside of demographic models specific to a system. This study presents a series of simulations utilizing demographic models that generalize biological scenarios of interest by borrowing population genetic parameters and genomic structure from humans and *Arabidopsis thaliana*, two markedly different organisms with markedly different population genetic parameters. We include realistic distributions of fitness effects and simulate under various models of dominance. In addition, we examine how the relationship between the genomic landscape of introgressed ancestry and recombination rates or functional content is determined by the underlying demography.

## Results

### Forward simulations

**Fig 1:**
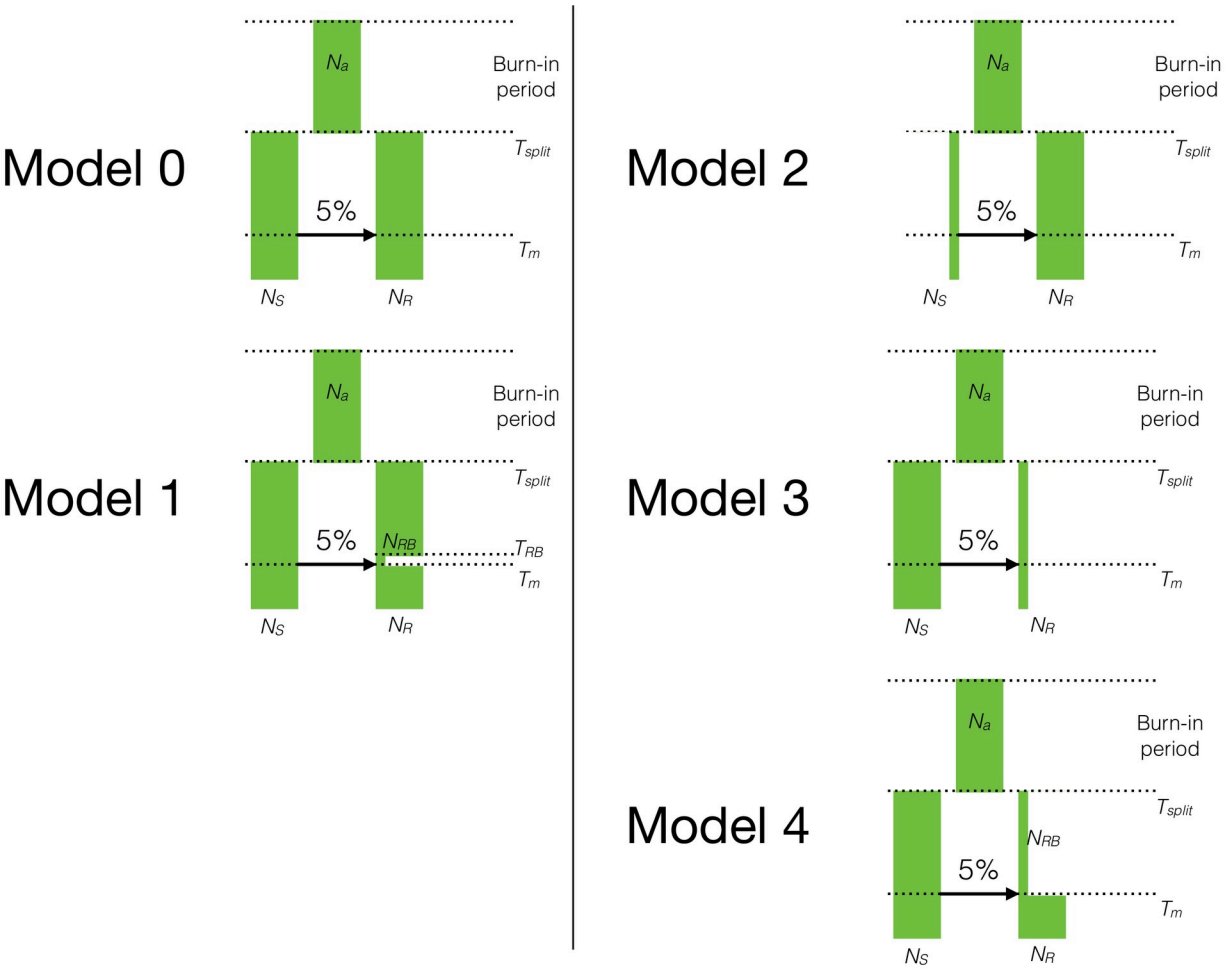
The demographic models used for the simulations. After a burn-in period of 10*N*_*A*_ (100,000) generations, a single population diverged into two subpopulations. The demography of the subpopulations was modified in ways that changed the distribution of deleterious variation. 2*N*_*A*_ (20,000) generations after the split, a single pulse of admixture occurred such that 5% of the ancestry of the recipient population came from the donor population. Arrows in each panel denote the direction of gene flow. The simulation was run for *N*_*A*_ (10,000) additional generations after admixture. Population sizes were changed as shown for each model. See S1 Table for specific parameter values for each model.

We used SLiM 3.0 [47] in conjunction with tools from pyslim [48] and msprime [49] to simulate a series of five models of admixture in the presence of deleterious variation. Each of the five models was based on a divergence model where an ancestral population at equilibrium splits into two subpopulations. At some time after the split, a single pulse of admixture occurs at a proportion of 5%, in one direction and for a single generation. Due to practical considerations only an initial admixture proportion of 5% was simulated. Fig 1 provides a cartoon representation of these models and the specific model parameters can be found in S1 Table.

All simulated sequence included genic structure (exon/intron/intergenic regions), which was either randomly generated or incorporated from a reference genome as described in the following sections. Only new nonsynonymous mutations were assigned nonzero selection coefficients, which were drawn from a gamma distribution of fitness effects (DFE) with shape parameter 0.186 and average selection coefficient E[*s*] = -0.01314833 [50] except when specified otherwise. In other words, no positively selected mutations were simulated.

Throughout, we will refer to the subpopulation that migrants originate from as the **donor** subpopulation, and the subpopulation that migrants join as the **recipient** subpopulation. Furthermore, we will refer to ancestry in the recipient subpopulation that originated in the donor subpopulation as **introgression-derived ancestry**. We use *p*_*I*_ to denote the total proportion of ancestry that is introgression-derived in the recipient subpopulation.

See the Methods for additional details on the simulations.

### Demography and recombination rate create differences in load between populations

To better understand how deleterious variants shape patterns of introgressed ancestry, we first simulated small genomic segments with randomly generated genic structure, of length ~5 Mb and selection coefficients from a gamma DFE. Two hundred simulation replicates using each of the 5 demographic models in Fig 1 (parameters in S1 Table), each of the per base pair recombination rates *r* = 10^−6^, 10^−7^, 10^−8^, and 10^−9^, and additive (*h* = 0.5) or recessive (*h* = 0.0) fitness effects were generated, for a total 8,000 independent replicates.

**Fig 2:**
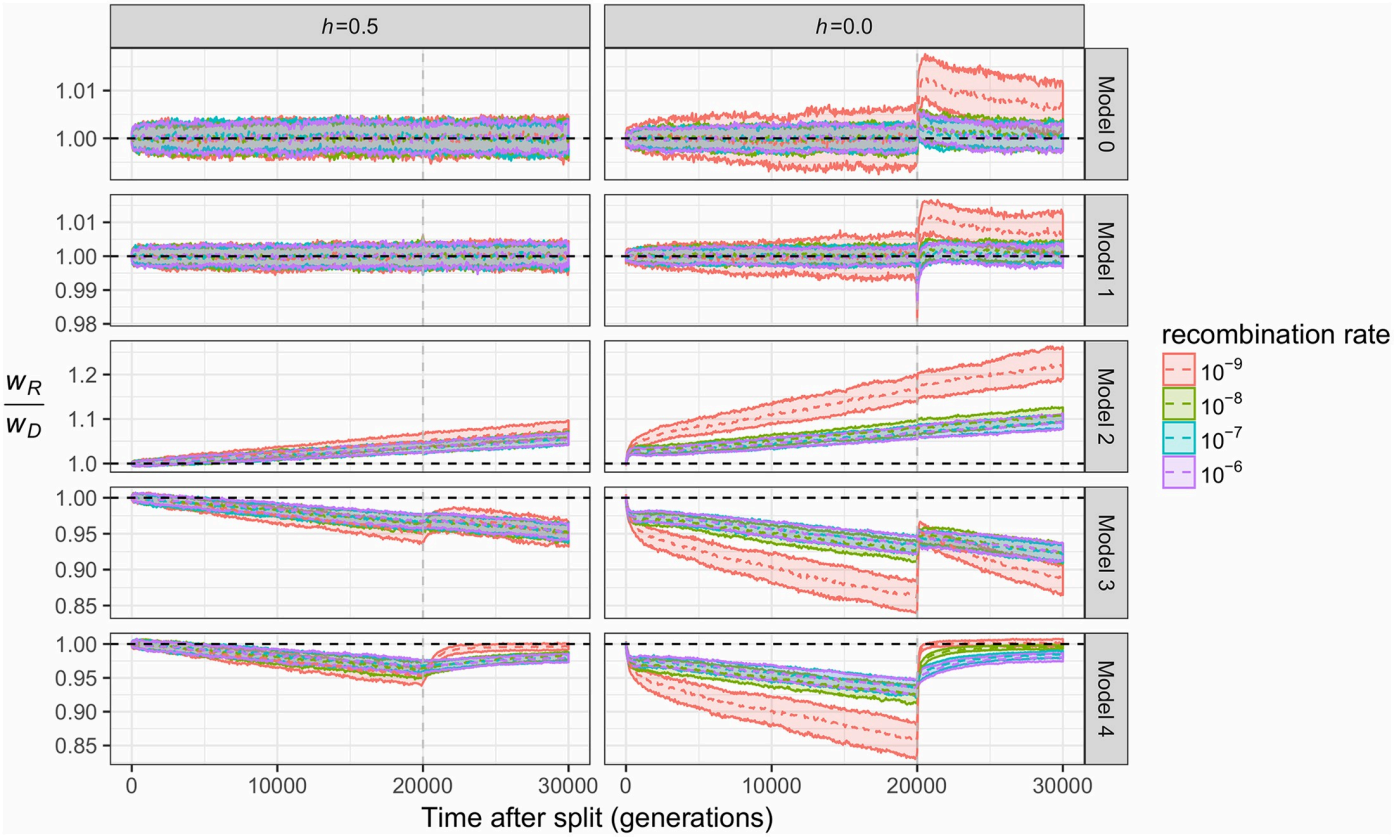
The change in the ratio of fitness over time due to demography. Each individual plot depicts the ratio of the mean fitness of the recipient population (*w*_*R*_) to the donor population (*w*_*D*_) for the demographic models shown in Fig 1. The mean (dotted line) and the 25^th^ to 75^th^ percent quantiles are shown for 200 simulation replicates. The vertical gray line depicts the time of gene flow, and the horizontal dashed black line depicts *w*_*R*_/*w*_*S*_ = 1. Different colors denote distinct recombination rates used in the simulations. Left panel denotes additive mutations (*h* = 0.5) while the right panel shows recessive mutations (*h* = 0).

In the 20,000 generations between the population split and admixture event, deleterious mutations accumulate at different rates across subpopulations for each unique model (S1 Fig), illustrated by the relative difference in subpopulation fitness in Fig 2. We report subpopulation fitness while ignoring the deleterious variants that have fixed in both subpopulations, since selection will not act on globally monomorphic variants. Because some weakly deleterious variants will fix in one subpopulation yet be lost in the other, each subpopulation’s fitness also steadily decreases through time.

In the additive fitness model, this relative difference in fitness is simply determined by relative differences in subpopulation size. When there are no differences in subpopulation size (Model 0), the fitness of both donor and recipient subpopulations decreases at approximately the same rate (*w*_*R*_ ≈ *w*_*D*_, Fig 2). A similar pattern is observed for a short bottleneck in the recipient population (*w*_*R*_ ≈ *w*_*D*_, Model 1, Fig 2), reflecting the insensitivity of additive genetic load (measured in terms of the number of deleterious variants per haplotype in S2 Fig) to short-term changes in *N*_*e*_ [9]. In contrast, long-term differences in population size (Models 2–4, Fig 2) provide enough time for deleterious variants to drift to higher frequency in the smaller subpopulation, resulting in substantial differences (approximately 5%) in fitness between subpopulations.

When deleterious mutations are recessive, a qualitatively similar relationship between subpopulation size and subpopulation fitness is generally observed. When there are no differences in population size (Model 0), the fitness of donor and recipient subpopulations decreases at a similar rate (*w*_*R*_ ≈ *w*_*D*_, Fig 2). A short bottleneck in the recipient population (Model 1) increases the frequency of homozygous, recessive genotypes immediately post-bottleneck (S3 Fig) which slightly decreases the recipient subpopulation’s fitness immediately before admixture (Fig 2). Finally, similar to the additive fitness model, long-term differences in population size result in substantial differences (>10%) in relative fitness between admixing populations.

The recombination rate is a key factor in determining differences in fitness between the two subpopulations. When the recombination rate is low, the fitness of the smaller subpopulation decreases more quickly, reflecting the reduced efficacy of purifying selection in low recombination regions [51]. Relative subpopulation differences in fitness between high recombination (*r* = 10^−6^) and low recombination (*r* = 10^−9^) simulations are about 2% for the additive fitness model and about 8% for the recessive fitness model.

### Demography and recombination determine changes in fitness post-admixture

Similar to the manner in which they affect subpopulation differences in fitness, recombination rates interact with demography to determine changes in subpopulation fitness after admixture.

When fitness effects are additive, admixture is unlikely to cause immediate and large changes in fitness, while subpopulation differences in fitness lead to gradual changes in fitness over time. If admixing subpopulations have the same fitness (Models 0 and 1, Fig 2 and S1 Fig), admixture predictably has no impact on the recipient population’s fitness. If donor haplotypes have lower fitness than the recipient (Model 2, Fig 2), the recipient population’s fitness is negligibly decreased by admixture (S2 Fig), specifically because relative differences in donor and recipient are small (<10%) and the initial frequency of donor ancestry is always 5%. If instead the donor subpopulation has higher fitness (Models 3 and 4, Fig 2), recipient fitness is relatively unaffected at the time of admixture but increases over time (S2 Fig) as the more fit haplotypes experience directional selection. The velocity and magnitude of these changes depends on the recombination rate, as variants originating from the same subpopulation are generally selected in the same direction, and these variants remain on the same haplotypes when recombination is low.

When fitness effects are recessive, admixture instead causes immediate and large changes in fitness as recessive alleles are masked in heterozygous, hybrid individuals (Fig 2 and S1 Fig). The qualitative patterns observed are consistent across all demographic models, but the magnitude of these changes is significantly larger in simulations where the recipient subpopulation has lower fitness. The recombination rate again plays a key role in determining fitness in the recipient subpopulation, with the largest changes in fitness occurring in simulations with low recombination. This occurs because the largest differences in pre-admixture fitness are observed when recombination is low (Fig 2), but also because the heterozygosity of hybrids is maximized if recombination does not occur between donor and recipient haplotypes. This linkage effect is particularly important as most of the variants under selection should have weak effects, since selection is likely to prevent strongly deleterious variants from drifting to high frequency even in a small population.

### Demography and recombination rate determine patterns of introgression

We next explore changes in the frequency of introgressed ancestry (*p*_*I*_) over time in the different models.

**Fig 3:**
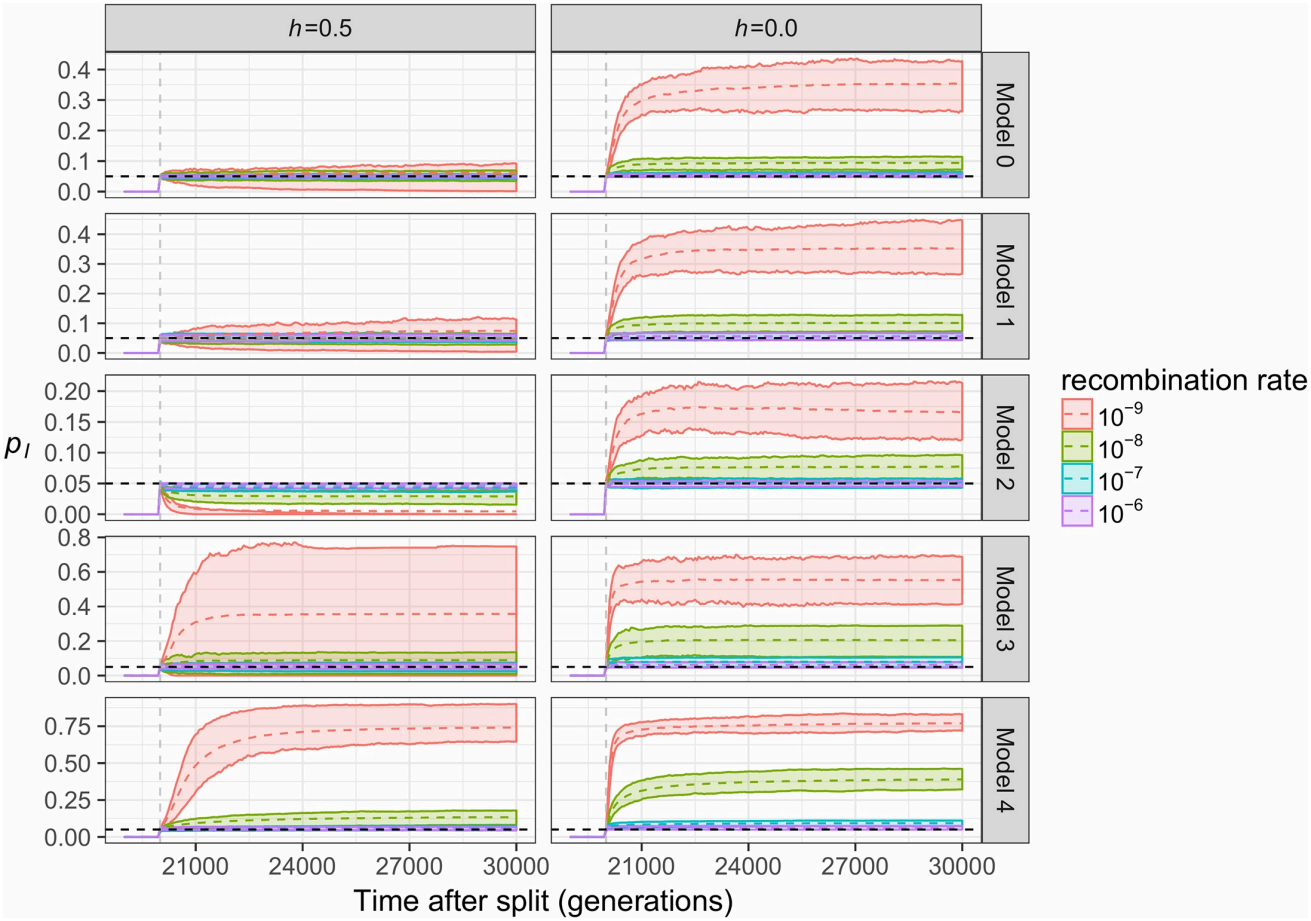
The frequency of introgression-derived ancestry (*p*_*I*_) in each model. Earlier generations are not shown since *p*_*I*_ = 0 prior to admixture. The mean (dotted line) and the 25^th^ to 75^th^ percent quantiles are shown for 200 simulation replicates. The vertical gray line depicts the time of gene flow, and the horizontal dashed black line depicts the initial admixture proportion of 0.05. Different colors denote distinct recombination rates used in the simulations. Left panel denotes additive mutations (*h* = 0.5) while the right panel shows recessive mutations (*h* = 0).

In the additive fitness case, changes in the frequency of introgression-derived ancestry are directly predictable from the differences in subpopulation fitness. When there are no differences in load (*w*_*R*_ ≈ *w*_*D*_, Models 0 and 1, Fig 2 and S1 Fig) between mixing haplotypes, selection does not favor a particular ancestry and donor subpopulation ancestry remains, on average, at the initial admixture proportion of 5% in the recipient (Fig 3). If donor subpopulation haplotypes have lower fitness as in Model 2 (Fig 2 and S1 Fig) deleterious donor ancestry is removed by selection, leading to a long-term *p*_*I*_ of less than 5%. If instead the donor subpopulation has higher fitness (Models 3 and 4, Fig 2), *p*_*I*_ is increased above 5% by selection. This increase is greatest (*p*_*I*_ = 75%) when there is an expansion after the time of admixture and in regions of low recombination (Model 4).

In a recessive fitness model, selection initially favors donor ancestry in the recipient subpopulation. In all cases (Models 0–4, Fig 3), the frequency of introgression-derived ancestry increases after admixture, regardless of whether the donor subpopulation’s fitness is less fit or more fit than the recipient. This effect is explained by heterosis, which occurs when recessive deleterious variants are masked as heterozygotes in hybrid individuals (S3 Fig), particularly in the generations immediately following admixture. At this time point, recombination has had little chance to shuffle donor and recipient haplotypes and heterozygosity is maximized in admixed individuals.

Again, the recombination rate is a key parameter that determines patterns of introgressed ancestry. As described previously, variants that are selected in the same direction remain linked when recombination is low (*r* = 10^−9^, Fig 3), maximizing the effect of selection and minimizing selective interference between recombinant haplotypes. When recombination is high (*r* = 10^−6^), the proportion of donor ancestry is unaffected by selection post-admixture (long-term *p*_*I*_ = 5%, Fig 3), as recombination quickly decouples variants under selection from their ancestry backgrounds. Importantly, when recombination rates are low (*r* = 10^−9^), the frequency of introgressed ancestry can increase substantially to up to 75% in the recipient population, despite the initial admixture proportion of 5%. Even with higher recombination rates, when deleterious mutations are recessive and there is a population expansion at the time of admixture (Model 4), introgressed ancestry can increase up to 25% frequency.

### The impact of the population split time on heterosis

So far, we have fixed the split time before admixture at 2*N* generations, a substantial time for differences in deleterious variation to accumulate between subpopulations. To further examine the relationship between split time and selection on introgression-derived ancestry, we simulated with Models 0 and 4 but also varied the time between the split and admixture (*t*_*s*_). For simulations with a demography analogous to Model 0, we simulated two divergent populations of equal size. For those analogous to Model 4, the recipient subpopulation’s size was reduced to 1,000 diploids immediately after the split and recovered to the original size at the same time that gene flow occurred. The recombination rate was set to *r* = 10^−9^ in these simulations.

**Fig 4:**
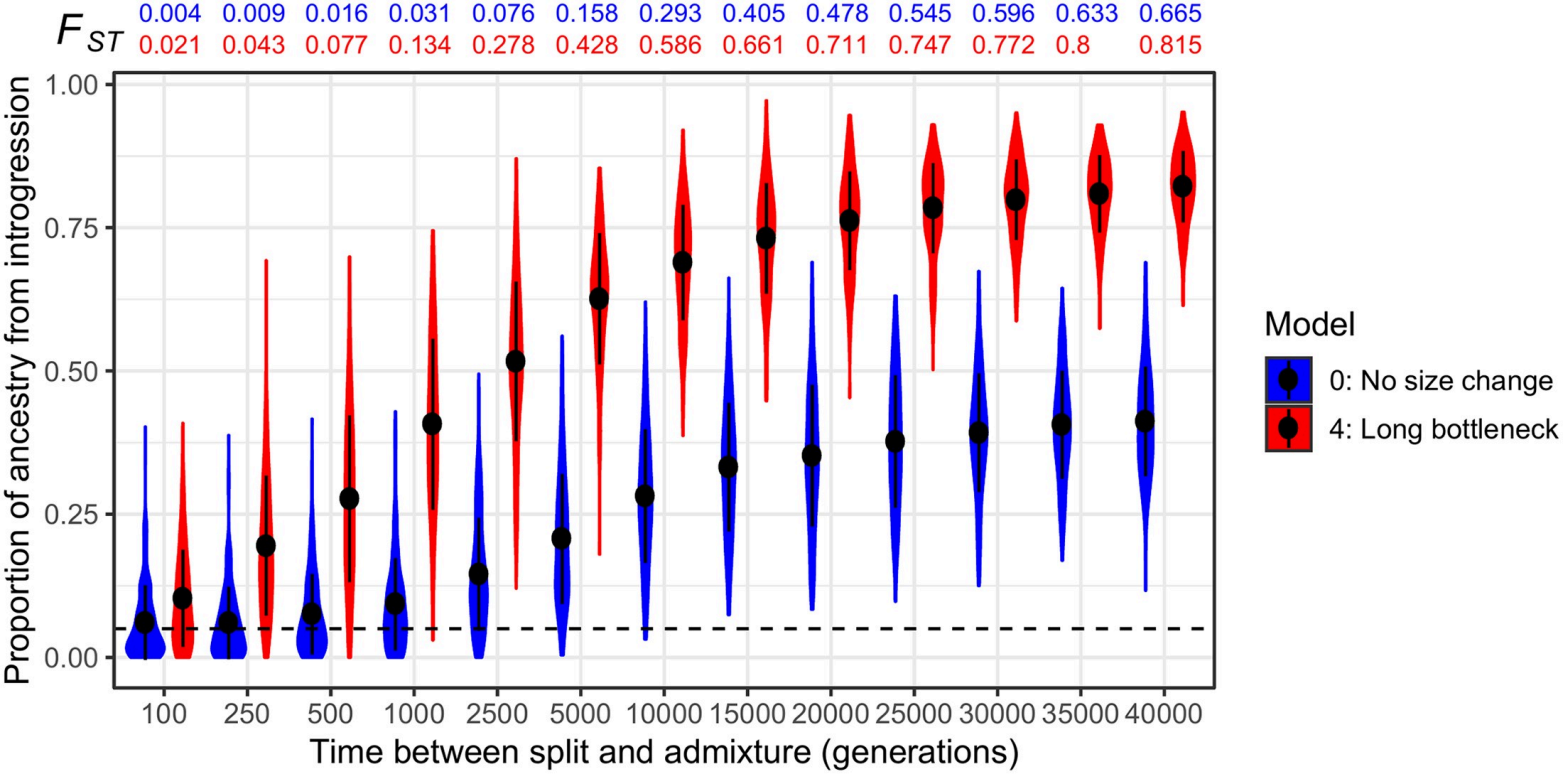
The effect of divergence and population size on introgression-derived ancestry when mutations are recessive. The proportion of ancestry that is introgression-derived, *p*_*I*_, at the time of *N*_*A*_ (10,000) generations after admixture, is shown for 200 simulation replicates and two demographic models (Model 0 and Model 4, refer to Fig 1) for a range of times between subpopulation divergence and the admixture event. The recombination rate in all simulations is *r* = 10^−9^ per base pair. Violin plots represent the density while dot and whiskers represent the mean and one standard deviation to either side. The horizontal dashed black line represents the initial admixture proportion of 0.05. Note that as the split time increases, *p*_*I*_ also increases. Values of *F*_*ST*_ reflect the amount of population differentiation at the split time, that is, immediately before admixture has occurs in each of these models.

Fig 4 depicts the long-term proportion of introgressed ancestry, *p*_*I*_, 10,000 generations after the admixture event for these two sets of models. We found that across our range of simulated *t*_*s*_, the long-term frequency of introgressed ancestry increases monotonically with *t*_*s*_ regardless of the underlying demography. Longer split times result in more deleterious variation being unique to each subpopulation, causing heterosis after admixture as private deleterious variants are masked by introgressed ancestry (S4 Fig). However, these differences appear to reach equilibrium after 20,000 generations (Fig 4), about when most deleterious variants are private to one subpopulation (Fig 4). We also found as a bottleneck increases in duration, differences in subpopulation fitness become a significant contributor to the increase in long-term *p*_*I*_, but note the apparent equilibrium at 20,000 generations. At a split time and thus bottleneck time of >20,000 generations, heterosis and differences in load increase long-term *p*_*I*_ nearly 2-fold relative the model with no differences in load (compare Model 0 to Model 4 in Fig 4). When parametrizing the population split times in terms of the realized *F*_*ST*_ values computed from the SNPs in the simulation output, we find that even for low levels of differentiation (*F*_*ST*_<0.05), there is a pronounced increase in introgressed ancestry (Fig 4). Interestingly, simulations with large long-term *p*_*I*_ (e.g. Model 4 at 1,000 generations or Model 0 at 5,000 generations) can have a level of differentiation of *F*_*ST*_<0.2 at the time of admixture, suggesting that even moderate levels of differentiation between subpopulations are sufficient to drive heterosis in low recombination regions (Fig 4).

### Human genome structure results in a heterogeneous landscape of introgression

So far, we have shown how selection on load shapes introgression-derived ancestry in a set of simple simulations. However, recombination rates and gene density are heterogeneous across actual genomes, and our simulations suggest this variation also could influence the genomic landscape of introgression.

To investigate how a realistic genomic structure affects patterns of introgression, we simulated with three of the demographic models described previously (Models 0, 2, and 4) using exon definitions and recombination map for a 100 Mb segment of human chromosome 1. We fixed the exon definitions and recombination map to be the same for all simulations. Only new nonsynonymous mutations were assigned non-zero selection coefficients drawn from a gamma DFE. In addition to simulating both additive and recessive fitness effects separately, we also simulated an inverse relationship between dominance coefficients and selection coefficients, which we will refer to as the *h*(*s*) relationship, using the function estimated by Henn et al. [14]. We generated 100 simulation replicates for each of the three demographic models. At the end of each simulation, we split the simulated chromosome into non-overlapping 100kb windows and computed the frequency of introgression-derived ancestry, exon density, and the average per base pair recombination rate in each window.

**Fig 5:**
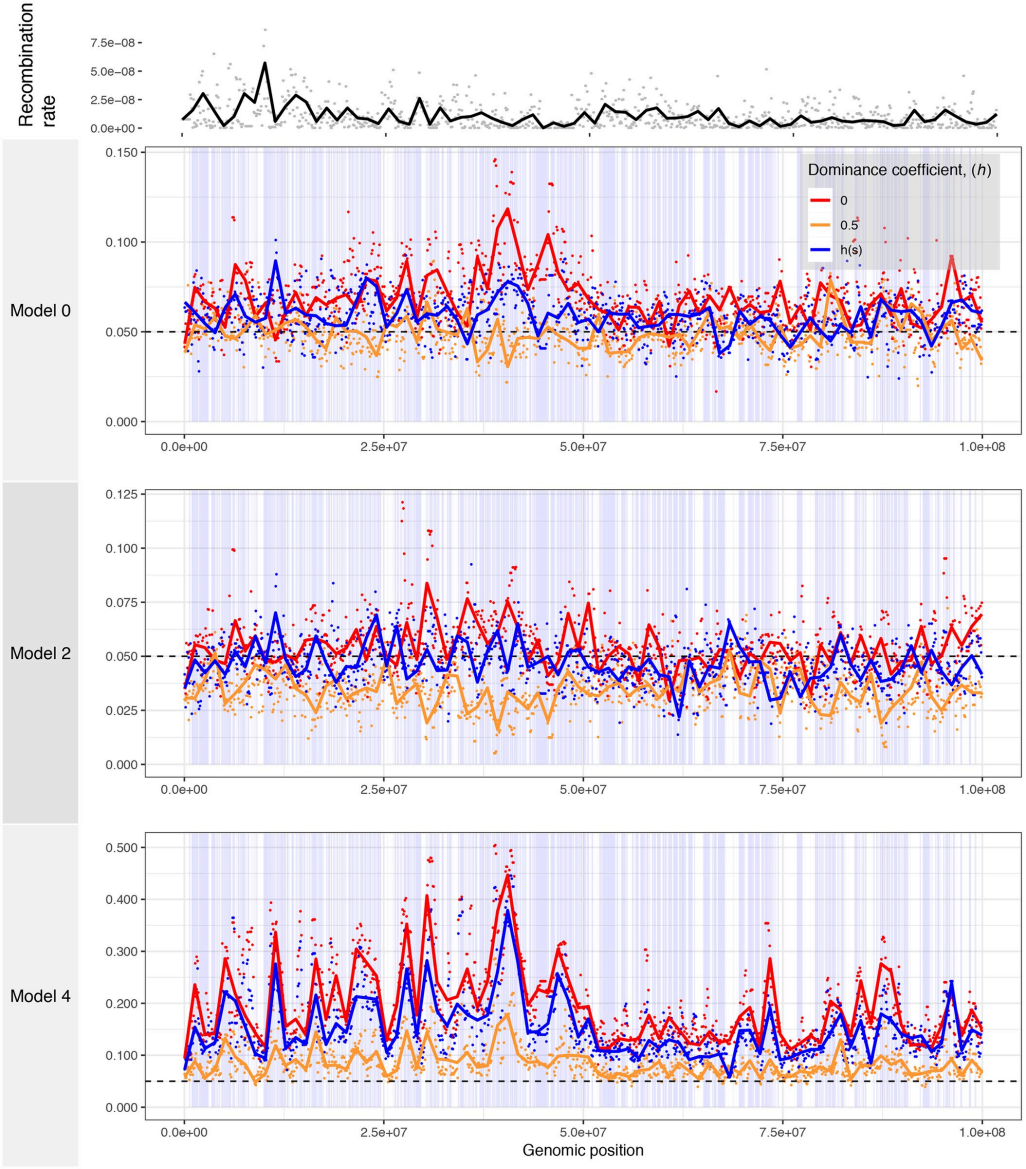
The average genomic landscape of introgression in simulations with human genomic structure. The frequency of ancestry that is introgression-derived is shown for non-overlapping 100 kb windows in a simulated 100 Mb region of chromosome 1. The model numbers refer to the models shown in Fig 1. Points represent a single value for each 100 kb window and lines are loess curves fitted to the data. The horizontal black dashed line represents the initial frequency of introgression-derived ancestry, *p*_*I*_ = 0.05. Vertical blue bars represent genes in which deleterious mutations can occur. Red curves denote the results for recessive mutations, orange curves show the results for additive mutations, and blue curves show the results for simulations with a *h*(*s*) relationship.

The frequency of introgression-derived ancestry generally exhibited genome-wide increases after admixture when mutations were partially or fully recessive and varied in accordance with differences in population size between subpopulations when mutations were additive. In the model with equal subpopulation sizes (Model 0), we observed no average change in the frequency of introgression-derived ancestry when mutations were additive. When new deleterious mutations were partially or fully recessive, we observed an overall genome-wide increase in the frequency of introgression-derived ancestry (Fig 5), with many regions reaching high frequency (>50%) in single simulation replicates (S5 Fig). This increase in frequency is only due to selection on recessive mutations and local variation in recombination rate, since no positively selected mutations were simulated.

In the model where introgressing haplotypes carried a larger deleterious burden (Model 2) and when deleterious mutations were not all recessive, we observed an overall depletion of introgressed ancestry consistent with the effects of purifying selection upon introgressed ancestry (Fig 5). However, in simulations with fully recessive mutations, the effects of heterosis were strong enough such that many genomic regions showed average increases in frequency of 1.5 to 2 times that of the initial introgression frequency of 5%. Importantly, Harris and Nielsen [18] predicted that heterosis would increase the frequency of introgressed ancestry by only a few percent, but our simulations with a similar demographic model show that larger increases in the frequency of introgressed ancestry are possible, especially in exon-dense and low recombination regions.

Finally, when we simulated the introgression of haplotypes from a subpopulation with lower genetic load (Model 4), we observed drastic, genome-wide increases in the average frequency of introgressed ancestry in the recipient subpopulations (Fig 5) as well as many fixed loci in individual simulations (S5 Fig), regardless of whether fitness effects of mutations were additive or recessive. For example, local regions of the simulated chromosome showed an average increase in introgressed ancestry from an initial frequency of 5% up to 50–60% frequency. Furthermore, peaks of introgression are highly correlated between the simulations with different models of dominance, suggesting that the interplay between exon density and recombination strongly affects the way that selection acts on introgressed ancestry in this model. This is the type of signature that is unlikely to be generated under neutral demographic models and could be mistakenly attributed to adaptive introgression.

It is also notable that the frequency of introgression-derived ancestry (*p*_*I*_) in each window appears to be driven not only by recombination but by exon density, or the local concentration of sites at which deleterious mutations can occur. For recessive mutations, *p*_*I*_ is greatly increased on the left-hand side of the simulated chromosome, which tends to be more gene-rich than the right-hand side of the chromosome (Fig 5 and S5 Fig). Importantly, the recombination rate was not significantly correlated with exon density (Spearman’s *ρ* = -0.0457, *p* = 0.149) in our simulations, showing these factors likely act independently to shape the landscape of introgression.

**Fig 6:**
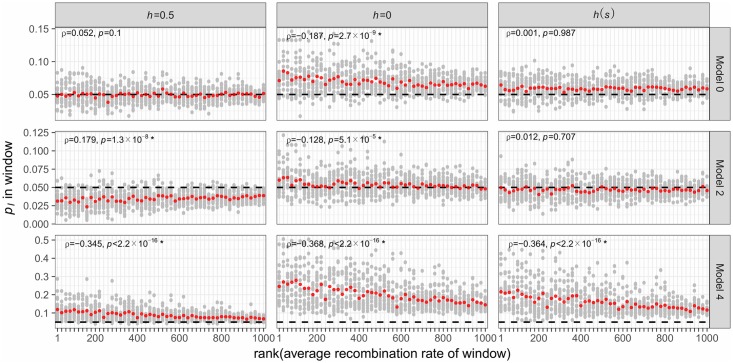
The relationship between recombination rate and introgression for different models of demography and selection. The frequency of introgression-derived ancestry (*p*_*I*_) is plotted against the ranked average recombination rate of non-overlapping 100 kb windows in each window at time *N*_*A*_ (10,000) generations after admixture. Gray dots represent the average *p*_*I*_ of a single window in 100 simulation replicates, while red dots represent the average *p*_*I*_ of 5% of windows as ordered by rank of recombination rate. Rank was randomly assigned for ties. The horizontal black line represents the initial *p*_*I*_ of 5%. Spearman’s *ρ* is computed for the relationship between recombination rate and *p*_*I*_ in each window and *p*-values indicate the significance of H_1_: *ρ*≠0. The model numbers refer to the models shown in Fig 1.

**Fig 7:**
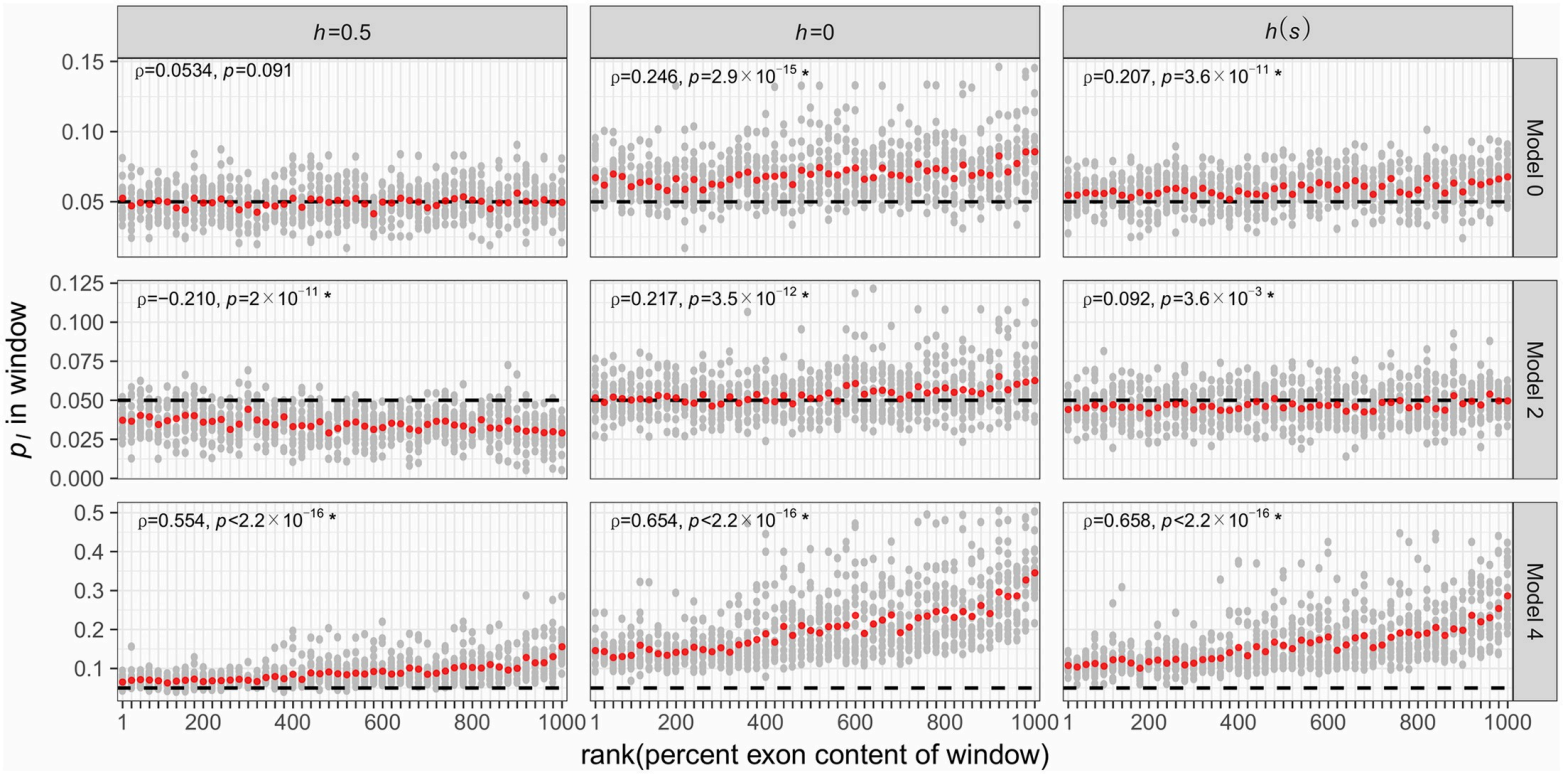
The relationship between exon density and introgression for different models of demography and selection. The frequency of introgression-derived ancestry (*p*_*I*_) is plotted against the average exon density of non-overlapping 100 kb windows in each window at time *N*_*A*_ (10,000) generations after admixture. Gray dots represent the average *p*_*I*_ of a single window in 100 simulation replicates, while red dots represent the average *p*_*I*_ of 5% of windows as ordered by rank of exon density. Rank was randomly assigned for ties. The horizontal black line represents the initial *p*_*I*_ of 5%. Spearman’s *ρ* is computed for the relationship between recombination rate and *p*_*I*_ in each window and *p*-values indicate the significance of H_1_: *ρ*≠0. The model numbers refer to the models shown in Fig 1.

To more formally explore these relationships, we examine the correlations between genomic features and the average frequency of introgressed ancestry across 100 simulation replicates, measured in 100 kb windows (Figs 6 and 7). In the model of equal subpopulation sizes (Model 0), the frequency of introgression-derived ancestry is not significantly related to the recombination rate or exon density when mutations have additive effects, but is positively correlated to exon density when fitness effects are fully or partially recessive (Fig 7). The *h*(*s*) relationship results in intermediate levels of introgression relative to simulations with strictly additive or fully recessive new mutations. For Model 2, the frequency of introgression-derived ancestry is positively correlated to the recombination rate and negatively correlated to exon density when fitness is additive. When fitness effects are fully recessive for this model, the frequency of introgressed ancestry is negatively correlated to recombination rate (middle panel in middle row in Fig 6) and positively correlated to exon density (middle panel in middle row in Fig 6). However, under the *h*(*s*) relationship, introgression derived ancestry is not significantly correlated to the recombination rate but is correlated with exon density. Lastly, when introgressed ancestry comes from a larger subpopulation with a lower deleterious burden than the recipient subpopulation (Model 4), the frequency of introgression-derived ancestry is always negatively correlated with recombination rate, and positively correlated with exon density. For Model 4, these correlations are observed for all models of dominance.

### Deleterious mutations impact the length of introgression deserts

Using these same simulations, we examined how selection on deleterious variation after admixture might influence the distribution of introgression deserts, or long stretches of the genome of the recipient population devoid of introgressed ancestry (S6 Fig). When subpopulation fitnesses are expected to be the same (Model 0), the distribution of introgression deserts for models with deleterious mutations is similar to a neutral model, suggesting that selection does not appreciably impact the distribution of deserts. When introgression-derived ancestry is expected to be deleterious (Model 2), simulations with additive fitness are enriched for longer ancestry deserts, though only slightly so. If instead introgression-derived ancestry is less deleterious than ancestry in the recipient population (Model 4), the length distribution of introgression deserts is shifted to be shorter, with the shortest introgression deserts occurring in models with recessive mutations (*h* = 0) where both selection on load and heterosis act synergistically to increase the frequency of introgressed ancestry.

### Introgression on the X chromosome

The observation that human X chromosomes are five-fold more resistant to introgression than the human autosome has been interpreted as a signature of genomic incompatibility between Neanderthals and humans, caused by an overrepresentation of male hybrid sterility genes on the X chromosome [24]. However, the evolution of the X chromosome differs from the autosomes in a number of important aspects, particularly in the strength of selection on deleterious variants [52], which may contribute to differences in patterns of introgression [18,19]. It is additionally unclear how selection on recessive variants might contribute, or counteract, the apparent resistance of the X chromosome to introgression.

To investigate the expected patterns of introgression on the X chromosome, we modeled X chromosome admixture with the simulation framework previously described. Although we used the same DFE for all these simulations, we utilized an analogous model of fitness that accounts for dosage compensation and the hemizygous sex [52,53]. Chromosome structure, recombination rates, and the DFE were the same as the simulations of human chromosome 1. See Methods for additional details on the calculation of fitness in these simulations.

**Fig 8:**
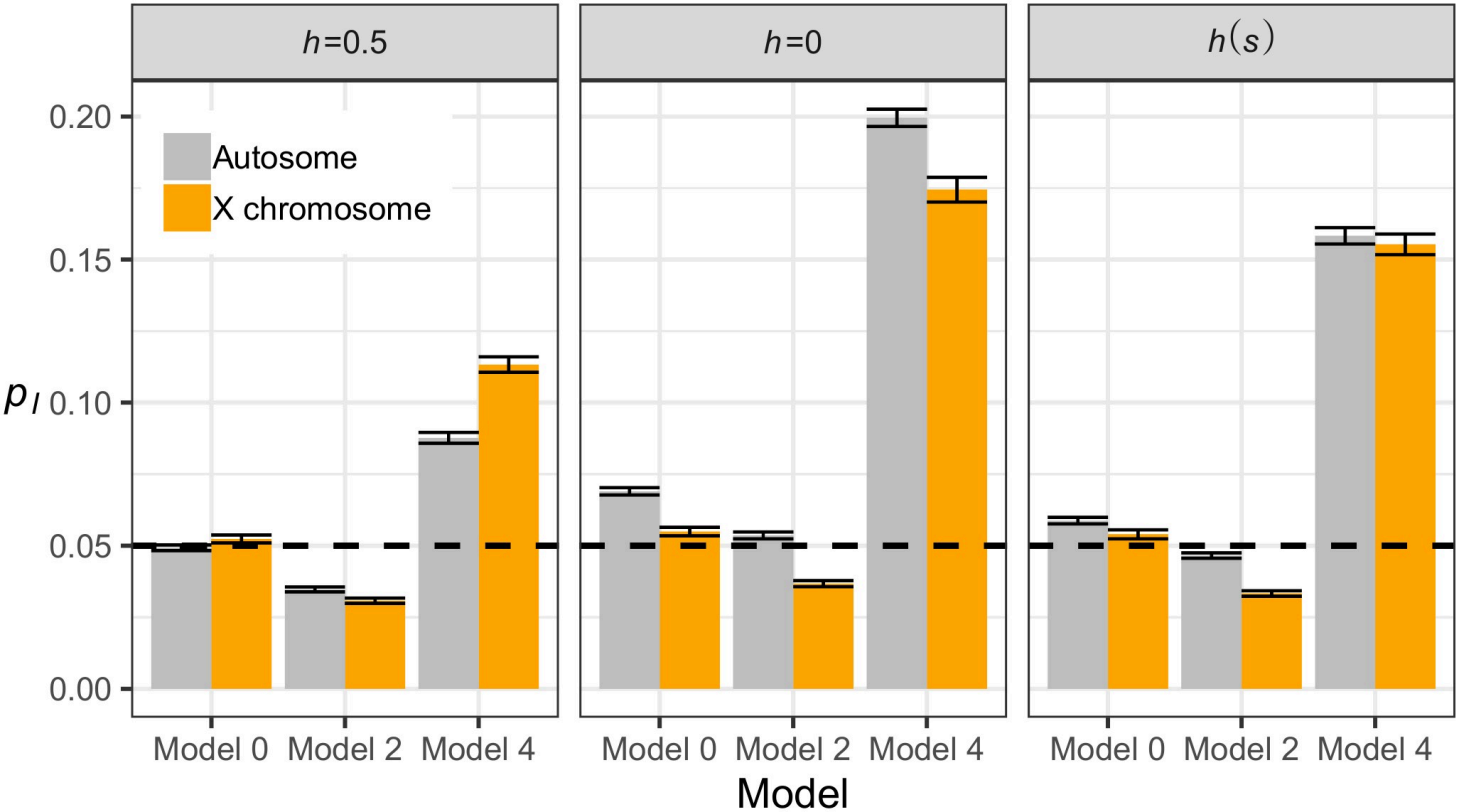
Differences in introgression between the X chromosome and autosomes. The average frequency of introgression-derived ancestry across the entire simulated chromosome (*p*_*I*_) at time *N*_*A*_ (10,000) generations after admixture is shown for three demographic models and three models of fitness. Model numbers refer to the models shown in Fig 1. Bars represent the mean *p*_*I*_ of 100 simulation replicates and error bars represent standard errors of the means. The horizontal dashed black line represents the initial *p*_*I*_ of 5%.

Our simulations show that deleterious variation alone can result in significant differences between introgression on the X and the autosomes (Fig 8). When fitness is additive, stronger overall selection occurs on the X chromosome because males cannot be heterozygous. This does not affect the X to autosome introgression ratio (X/A ratio) for Model 0, since both populations carry a similar burden of deleterious variants. For Model 2, selection removes introgressed ancestry from the X more quickly (X/A < 1), and for Model 4, selection increases the frequency of introgressed ancestry more on the X than on the autosomes (X/A > 1). When fitness is recessive, the effect of heterosis is weaker for the X chromosome, since the hemizygous sex cannot be heterozygous. This effect also results in less observed introgression on the X than the autosome (X/A < 1) for all considered models. Finally, under the *h*(*s*) relationship, our models predict amounts of introgression that are intermediate between strictly additive or strictly recessive models.

### Arabidopsis genome structure results in a homogeneous landscape of introgression

Human-like demography and genomic parameters may not generalize well for the purpose of understanding introgression in other species. Functional density, recombination rates, effective population sizes, dominance, and the DFE can differ by an order of magnitude between species. To provide an alternative picture of how introgression dynamics are driven by deleterious variation in a natural system where dominance and selection have been estimated, we simulated Models 0, 2, and 4 using the genomic structure of *Arabidopsis thaliana*.

While the simulated demography was similar to the ones described previously, we used exon definitions and a recombination map of most (29.1 out of 30.4 Mb) of *A*. *thaliana* chromosome 1, and chromosome structure was fixed to be the same in all 100 simulation replicates. Both exon density and recombination rates are higher in *A*. *thaliana* (medians of 100kb windows 4.8×10^−1^ and 3.2×10^-8^, respectively) than humans (medians of 100kb windows 1.6×10^−2^ and 8.04×10^−9^, respectively). The ancestral population size was set to *N*_*A*_ = 100,000 diploids, and the DFE to a gamma distribution with shape parameter 0.185 and E[*s*] = -0.0004866 [31]. We also assumed that dominance coefficients followed the *h*(*s*) relationship estimated by Huber et al. and did not simulate scenarios with only additive or only recessive new mutations. To the best of our knowledge, this is the only estimate of the *h*(*s*) relationship in a natural population other than humans. We split the simulated chromosome into non-overlapping 100kb windows and computed the frequency of introgression-derived ancestry, exon density, and the average recombination rate in each window.

**Fig 9:**
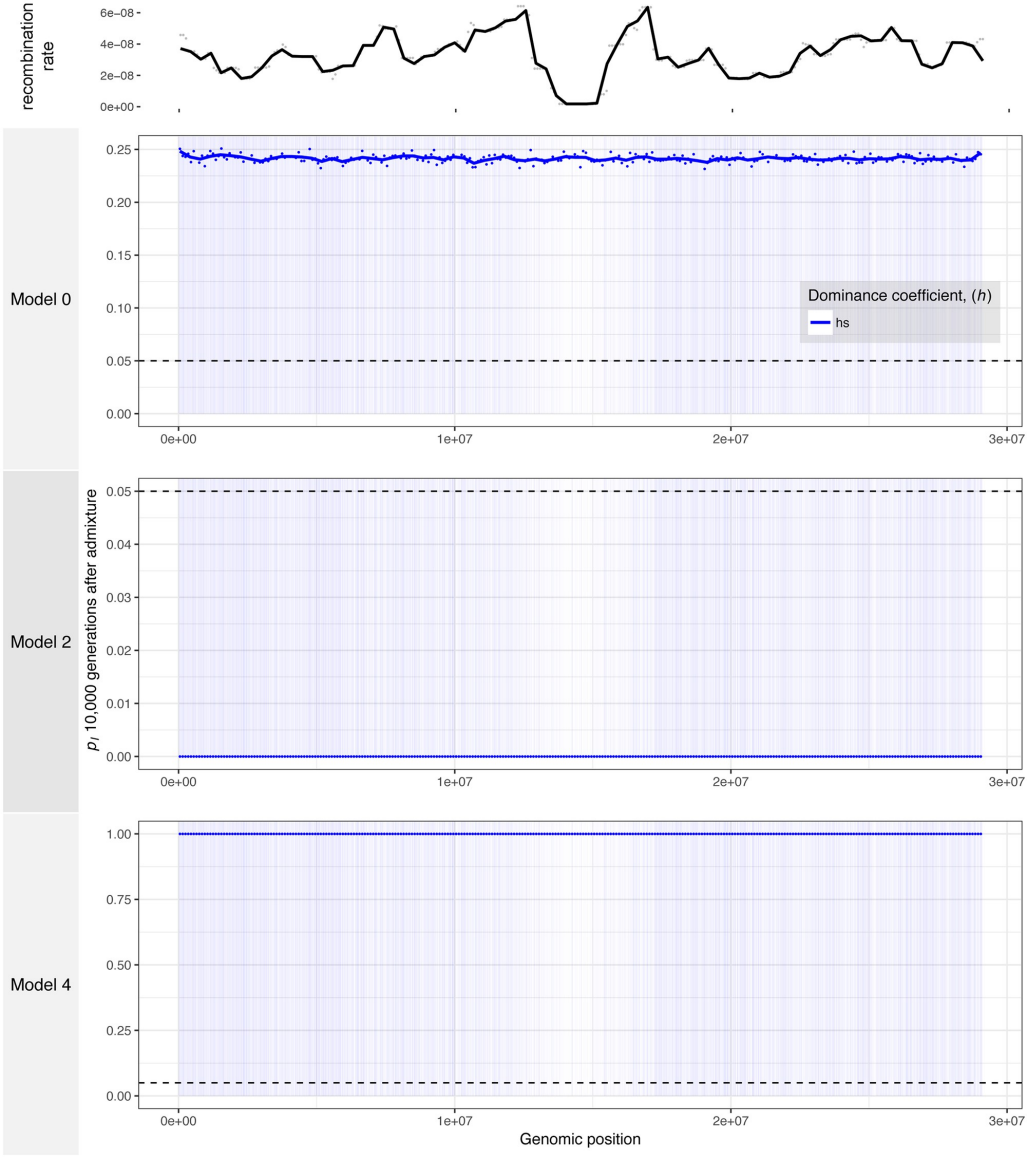
The average genomic landscape of introgression in simulations with *Arabidopsis* genomic structure. The frequency of ancestry that is introgression-derived is shown for non-overlapping 100 kb windows in a simulated 29.1 Mb region of chromosome 1. The model numbers refer to the models shown in Fig 1. Points represent a single value for each 100 kb window and lines are loess curves fitted to the data. The horizontal black dashed line represents the initial frequency of introgression-derived ancestry, *p*_*I*_ = 0.05. Vertical blue bars represent genes in which deleterious mutations can occur. Blue curves show the results for simulations with a *h*(*s*) relationship.

The genomic landscape of introgression in our simulated *Arabidopsis* population varied little (Fig 9), even in a single simulation replicate of the same demographic model (S7 Fig). For Model 0, introgressed ancestry rose quickly from an initial frequency of 5% to about 24%, *N*_*A*_ generations after admixture. There was little spatial variation in the frequency of introgression-derived ancestry. For example, *p*_*I*_ did not appear to be affected by the paucity of exons near the centromere (Fig 9). In Model 2, introgression-derived ancestry was quickly removed from the recipient subpopulation. This meant that *p*_*I*_ decreased to 0% across the whole chromosome. The converse was true for Model 4, where introgression-derived ancestry was favorable, and selection resulted in a complete replacement of recipient population ancestry (*p*_*I*_ = 100%).

### Introgression is more likely in partially selfing populations than outcrossing populations

A notable life history feature distinguishing *Arabidopsis thaliana* from its congeners is the capability to self-fertilize [54]. Populations that are capable of self-fertilization may experience an overall reduced *N*_*e*_ leading to an accumulation of weakly deleterious variants relative to an outcrossing population, and increased levels of inbreeding depression. On the other hand, strongly deleterious recessive mutations should be purged in a selfing population [55,56]. Relative differences in the types of deleterious variation between groups with different mating systems may then initiate another kind of selective tug-of-war after admixture.

**Fig 10:**
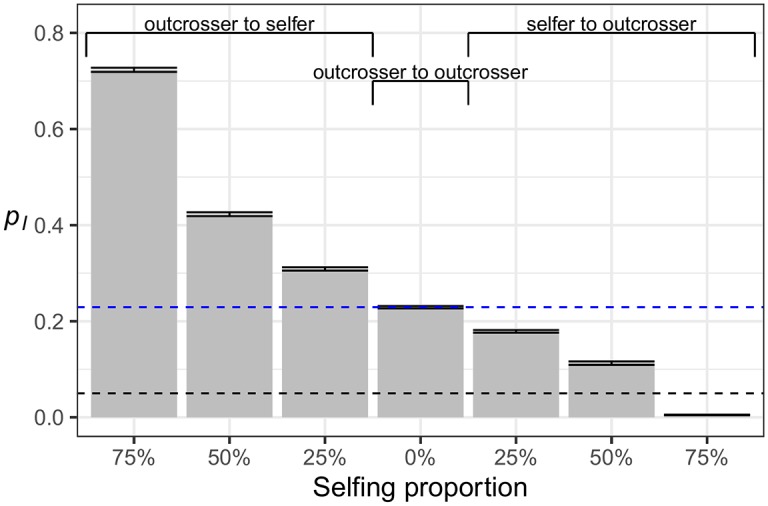
The impact of partial selfing on the frequency of introgression-derived ancestry. The simulated demographic model is Model 0 (Fig 1) with *Arabidopsis* genomic structure. The frequency of introgression-derived ancestry (*p*_*I*_) at time *N*_*A*_ (10,000) generations after admixture is plotted for seven different scenarios of admixture between a partially selfing population and an outcrossing population. Bar plots denote the average *p*_*I*_ of 100 simulation replicates and error bars represent standard errors of the averages. The horizontal dashed black line represents the initial *p*_*I*_ of 5%, and the horizontal dashed blue line represents the *p*_*I*_ that is expected when both subpopulations are outcrossers. Labels on the x-axis denote the probability of selfing in the population that is partially selfing.

To investigate how deleterious mutations affect levels of introgression when admixture occurs between two populations with different mating systems, we simulated gene flow between a partially selfing and an outcrossing subpopulation using the same *A*. *thaliana* parameters as described in the previous section. We limited our simulated demographic model to Model 0 so that any difference in deleterious variation between subpopulations could be attributed to the mating system. Seven different gene flow scenarios were simulated, with selfing probabilities of 0%, 25%, 50%, and 75% in either subpopulation (Fig 10). Specifically, we simulated: first, with two outcrossing populations (0% to 0%); then with the outcrosser (0%) as the donor and the partial selfer (selfing probabilities of 25%, 50%, 75%) as the recipient, then the partial selfer (25%, 50%, 75%) as the donor and the outcrosser (0%) as the recipient. Self-incompatibility alleles were not simulated.

Our simulations show that the long-term frequency of introgression (10,000 generations after admixture) depends on the proportion of selfing individuals in the selfing subpopulation (Fig 10). In other words, selfing reduces *N*_*e*_ relative to an outcrosser, resulting in increased drift and a greater accumulation of deleterious mutations. These differences in load result in patterns of introgression qualitatively similar to those observed previously in this study. In the simulations between two outcrossing populations, *p*_*I*_ increases from 5% to a long-term 20–25%, due to heterosis from the large proportion of recessive mutations predicted by the *h*(*s*) relationship. This is the same result as the simulations of Model 0 in the previous section. When the outcrosser is the donor, *p*_*I*_ increases monotonically with the selfing probability of the recipient, this time above the fraction expected between two outcrossing populations. When the partially selfing population is the donor, long-term *p*_*I*_ usually increases by heterosis from the initial 5% value, although the long-term *p*_*I*_ monotonically decreases as the selfing probability increases. At a selfing probability of 75%, the outcrossing population is almost completely resistant to introgression. In the absence of fitness epistasis, it is likely that a combination of high recombination rates and strong initial selection from differences in deleterious mutations between populations counteracts any loss of donor ancestry from the purging of strongly deleterious recessive variants.

## Discussion

We have shown through simulations that deleterious variation can greatly influence the dynamics of introgression between admixing populations, in markedly different directions, magnitudes, and manners depending on the demographic model, mating system, models of selection, and genomic structure. In particular, the recombination rate is a key parameter that determines the way in which deleterious variants accumulate between populations and how selection acts on introgression-derived ancestry after admixture, ultimately determining the genomic landscape of introgression.

Our work demonstrates how demography can shape patterns of deleterious variation in different populations. Previous studies have examined the role of population size changes [1,2,9,13,57] and serial founder effect models [14,58] on deleterious variation. Interpreting how differences in the distribution of deleterious variation impact fitness has been a contentious issue [5,6,8–10,12]. In this study, we observed that admixture can increase the fitness of the recipient population, sometimes drastically if the donor population is of larger long-term effective population size and thus carries lower genetic load. Generally, gene flow is observed to drive smaller, subtle changes in fitness. Nevertheless, the influx of new alleles can result in a rearrangement of deleterious variants in an admixed population (S2 and S3 Figs), and subtle changes to fitness can lead to significant shifts in the frequency of introgressed ancestry (e.g. see Model 0, *h =* 0.0, in Fig 3). These effects can be long lasting, persisting for thousands of generations in some of our simulations (Figs 2 and 3, S1 Fig). If hybridization is a significant feature of a study population, studies concerning load should consider the fitness consequences of admixture as well as population size changes.

That dynamics of introgression-derived ancestry can be driven by deleterious variation is also important for the study of selection on gene flow between populations or species. Patterns of introgression between hybridizing species are often asymmetric, vary across the genome, and can be driven by demography at expansion fronts [59], dispersal processes [60], or by natural selection. However, when natural selection is implicated as driving changes in introgression-derived ancestry, processes such as genomic incompatibility or adaptive introgression are invoked to explain variation in introgression across the genome. We have shown that differences in demography and mating system create between-population differences in standing deleterious variation, and that selection upon these differences provides an alternative hypothesis to selection on alleles transplanted onto a new genomic background or new environment. To the best of our knowledge, only a few studies have considered the contribution of selection on deleterious variation to observed patterns of introgression [13,25,33], and mostly in specific systems [18,19,23,26].

Selection on deleterious variation may be particularly important for determining patterns of introgression in natural populations that are out of demographic equilibrium. Models of increased genetic drift predict accumulations of genetic load at the edges of expanding populations [14,58] which suggests introgression into the expanding population could be driven by selection on deleterious mutations. We have also shown that population bottlenecks can greatly affect patterns of introgression, particularly when assuming a recessive fitness model. If recessive deleterious variation also creates heterosis in admixed individuals, the effects of heterosis and population size will be synergistic, further enhancing introgression in genomic regions of low recombination. Our simulations also directly suggest heterosis may contribute to the pervasive patterns of introgression and shared polymorphism between different species in the genus *Arabidopsis* [36] even if hybridizing species have similar amounts of deleterious variation.

Because selection can alter patterns of introgression even if hybrid ancestry is not explicitly deleterious, genome-wide inferences of admixture proportions that assume neutrality are likely to be biased. For instance, our simulations predict the amount of introgression is strongly influenced by deleterious mutations in Arabidopsis, and the manner in which this occurs is dependent on the demography. Observed proportions of ancestry range from 0% for Model 2 to 100% for Model 4 (Fig 9 and S8 Fig), despite the true admixture proportion of 5%. Taking the observed proportion of introgressed ancestry at face value, researchers would not infer the true initial admixture proportion of 5% accurately. Similarly, linkage disequilibrium patterns are often used to infer the timing of admixture events and to test competing demographic hypotheses about admixture [61]. If the distribution of segments of introgressed ancestry can be altered by deleterious mutations relative to what is predicted under a neutral model (e.g. Model 4 in S7 Fig), these inferences can also be biased. To circumvent this problem, we recommend focusing on putatively neutral regions of the genome far from genes.

Likewise, our simulations may provide grounds for a plausible alternative explanation for the negative correlation between recombination rate and introgressed African ancestry observed in North American populations of *D*. *melanogaster* [44,45], which is the opposite of what is usually observed by other empirical studies of hybridization. Corbett-Detig and Nielsen [45] proposed that widespread adaptive introgression could bring along larger linkage blocks in low recombination regions. If *D*. *melanogaster* has accumulated genetic load through the serial colonization of the world in association with humans [62,63], selection may favor introgression of the origin population (African) haplotypes in low recombination regions, similar to what we observed in Model 4 of our simulations. This could act synergistically with the effect of heterosis, which can happen in significant amounts even when divergence is low (Fig 4), and the divergence for which significant increases in introgressed ancestry are observed is comparable to that between populations of *D*. *melanogaster* [64]. Admittedly, our models bear little resemblance to the estimated demography of *D*. *melanogaster* (e.g. [63]). Similar to humans [9], there may be little difference in additive load between populations due to recent demography, and we have not simulated with a DFE and model of dominance estimated from *D*. *melanogaster*. Further study of these population genetic features is necessary to estimate the relative contribution of these processes to the genomic pattern of introgression in *D*. *melanogaster*.

Importantly, we do not claim that deleterious variation can explain all the patterns of introgression in any species, but rather that it is a plausible alternative explanation and therefore possible confounder that is important to consider when testing hypotheses about the nature of selection on gene flow. It is alternatively possible that colonizing populations of *D*. *melanogaster* experience a reduction in the rate of fixation of adaptive alleles due to reduced *N*_*e*_, creating favorable conditions for the introgression of parent population haplotypes. Additionally, there is strong evidence for the role of sexual selection and fitness epistasis between the X and the autosomes in separating populations of *D*. *melanogaster* [65–67]. In hybridizing swordtail fish, recombination rates are positively correlated with the frequency of introgressed ancestry even when the minor parent population, analogous to the donor population in our simulations, has a larger effective population size [26]. This pattern suggests that hybrid ancestry has an overall deleterious effect, meaning that genomic incompatibility is the dominant force shaping hybrid genomes in that system. In humans, regions of high recombination rate are enriched for introgressed Neanderthal ancestry particularly in genes that code for virus-interacting proteins [43], suggesting that in these regions putatively adaptive variants were more likely to recombine off the deleterious Neanderthal background and increase in frequency. In these two latter cases, selection on deleterious variation or heterosis may instead obscure genome-wide signals of incompatibility or adaptive introgression.

Because selection on additive and recessive variation can act in complementary or opposing directions, our study also highlights the fundamental importance of understanding the distribution of selection coefficients and their relationship to dominance coefficients in natural populations (i.e. the *h*(*s*) relationship). In this study, we simulated human genomic structure, where new mutations are more likely to have additive fitness effects [14], and *Arabidopsis* genomic structure, where deleterious new mutations are likely to be more recessive [31]. In these two scenarios, we found that modes of dominance interacted with demography, recombination rates, and functional density in complex ways. Importantly, we observed an increase in introgressed ancestry as a result of the heterosis effect even when mutations were not completely recessive, that is, dominance was modeled with the *h*(*s*) relationship. While the effects observed in the present study may be applicable to real populations with realistic amounts of dominance, the *h*(*s*) relationship is unknown for virtually all natural systems. Therefore, we cannot easily predict the contribution of heterosis to introgression and shared polymorphism between closely related species.

Nevertheless, the underlying demographic model will determine how additive and recessive new mutations should interact after gene flow. For example, the introgression of deleterious haplotypes in Model 2 was facilitated by heterosis but impeded by additive load, leading to uncertainty about the overall contribution of the effects of deleterious variation in certain scenarios, such as Neanderthal to human admixture [18]. In other demographic models, selection on additive and recessive variants should operate in the same direction. As another example, if admixture occurs between a partially selfing and outcrossing population, our simulations predict that selection works to remove ancestry from the selfing population, since it carries an overall larger burden of deleterious variants. It may yet be possible that strongly deleterious recessive variants, which should be purged in the selfer, play a role in preventing some introgression from the outcrossing to the selfing population. Without knowing the *h*(*s*) relationship for a specific system, it is difficult to disentangle the effects of selection on additive versus recessive variation.

Our work further highlights the importance of considering deleterious variation when comparing complementary lines of evidence to make inferences about selection on hybrids. Even in the absence of fitness epistasis, our models predict an overall depletion of hybrid ancestry on the X chromosome compared to the autosomes. While the magnitude of this difference (about 1.5-fold) is far less than the 5-fold difference observed in humans [24], our results clearly show that simpler models of deleterious variation have the potential to mimic some of the signals that are considered evidence of hybrid incompatibility. Granted, we have only provided a simple model of selection on sex chromosomes to contrast to previous simulations of the autosomes, while ignoring the fact that recombination, chromosome structure, and the DFE are unlikely to be the same between the X and the autosomes. Additionally, it has been shown that sex-biased demographic processes have occurred throughout human history [68–72]. Future work should test the extent to which our results hold across more realistic population genetic models.

The recombination rate also plays a key role in determining the landscape of introgressed ancestry in the presence of deleterious variation. Models of Hill-Robertson interference [51,73] predict that deleterious mutations will not be removed as effectively in regions of the genome with low recombination rates when weakly selected mutations occur on different haplotypes, since selection on a particular site will weaken selection (i.e. increase drift) at other linked sites. We observe this effect in our simulations, where fitness declines the fastest when recombination rates are low, both pre- and post- admixture (S2 Fig). However, we observe the opposite effect immediately after admixture. Specifically, in our simulations, the fitness in the admixed population increased the most for the lowest recombination rates, suggesting that deleterious mutations were most effectively eliminated when recombination rates were the lowest (S2 Fig). This occurs because selection for a haplotype will be most effective when all alleles on a haplotype tend to have weak fitness effects in the same direction [18,19,26]. For example, if introgression-derived ancestry carries fewer deleterious variants than the other haplotypes in the recipient population, selection will act to increase the frequency of the protective alleles contained within the introgressed ancestry. This applies directly to our simulations of admixture since immediately following an admixture event, all the protective or deleterious variants are found on the same haplotype. Higher rates of recombination will shuffle selected variants onto different haplotypes, creating selective interference between recombinant haplotypes.

One significant limitation of our study is that we have not considered all possible combinations of demographic, selective, and genomic parameters relevant for all species. For example, heterosis appears to stabilize long-term patterns of introgression at some frequency, but we only simulated an admixture fraction of 5%. It is possible that the magnitude or direction of observed changes may change with different major and minor parent ancestry proportions. It is therefore difficult to directly assess whether the specific conclusions seen for one combination of parameters will directly apply in a different specific system. Instead, our goal is to highlight the need to consider deleterious variation as a possible null model that should be investigated and rejected before attributing unusual patterns of introgressed ancestry to other evolutionary processes. That being said, we have observed some commonalities across models. For example, in Model 4, when mutations are either fully recessive or have an intermediate dominance coefficient assigned as a function of the selection coefficient, we observe an increase in introgressed ancestry in the recipient populations when either using simple models (Fig 3), models relevant for human populations (Fig 5) or models relevant for *A*. *thaliana* (Fig 9).

This interplay between deleterious variation and recombination has substantial implications for detecting adaptive introgression. A major objective of genomic studies of hybridization is to identify loci that are adaptively introgressed and to ascertain the overall importance of introgression to adaptive evolution [38]. Genomic regions that contain introgressed haplotypes at high frequency are considered likely candidates for adaptive introgression [38,41,74,75], but we have shown that selection on deleterious mutations can increase the frequency of introgression-derived ancestry, even in the absence of beneficial new mutations. Thus, outlier-based approaches that compare summary statistics computed for a particular window of the genome to a null distribution that does not account for deleterious variation may be misled. Linked deleterious variants may also impede positive selection on introgressed adaptive variants, particularly if they are recessive [76]. Because recombination can move an adaptive variant off of ancestry backgrounds of varying fitness, standard models of adaptive evolution, especially ones that do not consider deleterious variation, are unlikely to accurately describe genomic patterns generated by adaptive introgression. Finally, it may be difficult to differentiate heterosis due to the masking of deleterious recessive alleles from heterozygote advantage at introgressed loci, despite the fact that these are two very different evolutionary processes with dramatically different biological interpretations.

Our results argue that new null models are needed in studies seeking to identify candidates of adaptive introgression. These new null models should include deleterious genetic variation, as well as complex demography. In order for these models to accurately capture the dynamics of deleterious variation, they should also include realistic parameters for the DFE of deleterious mutations and the relationship between dominance coefficients and selection coefficients. Lastly, the new null models should also include realistic models of the variation in recombination rate across the genome, as recombination rate is a key determinant of the dynamics of introgression (Fig 3). Failure to consider deleterious variation in a realistic way in studies of admixing populations or hybridizing species can mislead inferences about the evolution of hybrids.

## Materials and methods

### Simulation details

All simulations were performed with SLiM 3.0 [47]. We chose to discard from our simulations, and therefore from calculations of fitness, mutations that were fixed in the ancestral or both subpopulations. Although fixed deleterious variants contribute to the overall genetic load of finite populations, they will have no effect on the relative differences between admixing subpopulations and no effect on the dynamics of introgression-derived ancestry. Therefore, each fitness calculation does not reflect the true fitness of each population, but rather the fitness components that are relevant during gene flow.

An admixture event in SLiM is handled by modifying the way the parents in each generation are chosen (SLiM manual 5.2.1). For example, at an admixture proportion of 5% the recipient population reproduces as follows. Five percent of the parents of the recipient population, in that generation, are chosen from the donor population, and 95% of the parents are chosen from the recipient population.

### Scaling of forward simulations

We rescaled simulation parameters by a scaling constant, *c*, to reduce the computational burden of forward simulations. Population sizes were scaled to be *N*/*c*, times to *t*/*c*, selection coefficients to *sc*, and the mutation rate to *μc*. Recombination rates were scaled as 0.5(1-(1-2^*r*^)^*c*^), which is approximately *rc* for small *r* and small *c*. The total length of simulated sequence was not changed in scaled simulations. Note, the simulation parameters we reference in this paper are always unscaled. The manner in which we scaled simulations follows Algorithm 1 in Uricchio and Hernandez [77] and is similar to how Lange and Pool [78] simulated populations of *Drosophila melanogaster*, although the primary features of interest in our simulations are related to the dynamics of introgression-derived ancestry through time.

Because scaled simulations may not exactly recapitulate the dynamics of unscaled simulations, we used a set of test simulations to choose *c* = 5 for most simulations. The dynamics of *p*_*I*_ for scaled simulations (*c* = 2, 5, and 10) were compared to an unscaled simulation (*c* = 1), using the demography of Model 4, a gamma DFE, and additive fitness (*h* = 0.5). Per base pair recombination rates of *r* = 10^−7^ and 10^−8^ were simulated separately. Although all scaled simulations exhibit slight differences from the unscaled simulations, a scaling factor of *c* = 5 provided a reasonably accurate representation of the unscaled dynamics of *p*_*I*_ (S8 Fig) while keeping simulation run times within reasonable limits. We additionally note that our intent in this study is to understand qualitative patterns of introgression rather than to obtain accurate quantitative estimates from a particular system, and the qualitative patterns are consistent irrespective of the scaling factor.

### Tracking introgression

The proportion of admixture that is introgression-derived (*p*_*I*_) was tracked in one of two ways: by placing marker mutations at a fixed interval or by tracking the tree sequences (genealogies) across the simulated genome. In the former case, *p*_*I*_ was estimated by placing marker mutations in the donor population immediately before the admixture event. These mutations were spaced at 500 base pair intervals over the genome of every individual. After admixture, *p*_*I*_ was estimated in the recipient population by taking the averaged allele frequency of marker mutations per window, or throughout the whole simulated chromosome. In the latter case, the true ancestry proportions were calculated, since the information on start/end coordinates and the lineages that trace their ancestry back through donor and recipient populations is preserved. Although tracking tree sequences provides the most accurate estimate of *p*_*I*_, marker mutation tracking was used for computational efficiency in some simulations.

### Simulations with randomly generated chromosomal structure

The sequences from simulations with randomly generated chromosome structure were approximately 5Mb in length, and contained intergenic, intronic, and exonic regions, but only nonsynonymous new mutations experienced natural selection. The per base pair mutation rate was constant and set to *μ* = 1.5×10^−8^ and we set nonsynonymous and synonymous mutations to occur at a ratio of 2.31:1 [79]. The selection coefficients (*s*) of new nonsynonymous mutations were drawn from a gamma-distributed DFE with shape parameter 0.186 and expected selection coefficient E[*s*] = -0.01314833 [50] for both additive and recessive dominance models.

The chromosomal structure of each simulation was randomly generated by drawing exon lengths from *Lognormal*(*μ* = *log*(50), *σ*^2^ = *log*(2)), intron lengths from *Lognormal*(*μ* = *log*(100), *σ*^2^ = *log*(1.5)), and the length of noncoding regions from *Unif*(100,5000), following the specification in the SLiM 3.0 manual (7.3), which is modeled after the distribution of intron and exon lengths in Deutsch and Long [80]. The per base pair per chromosome recombination rate (*r*) was fixed in each simulation, but we varied *r* between different sets of simulations where *r* ∈{10^−6^,10^−7^,10^−8^,10^−9^}. Lastly, we simulated 200 replicates for each set of simulations, of each specific *h* and *r*.

Chromosome-wide *F*_*ST*_ was calculated for all variants from exons, introns, and intergenic regions by calculating *F*_*ST*_ at individual sites following Hudson et al. [81] and by combining *F*_*ST*_ across sites following Bhatia et al. [82].

### Simulations of human genomic structure

In simulations of fixed chromosome structure from the human genome, we fixed the structure to 100 Mb randomly chosen from human genome build GRCh37, chromosome 1 (chr1:5,005,669–105,005,669). The exon ranges were defined by the GENCODE v14 annotations [83] and the sex-averaged recombination map by Kong et al. [84], averaged over a 10 kb scale. The per base pair mutation rate was constant and set to *μ* = 1.5×10^−8^ and we set nonsynonymous and synonymous mutations to occur at a ratio of 2.31:1 [79]. The selection coefficients (*s*) of new nonsynonymous mutations were drawn from a gamma-distributed DFE with shape parameter 0.186 and expected selection coefficient E[*s*] = -0.01314833 [50] for all models of dominance. All other new mutations were neutral. We simulated additive fitness (*h =* 0.5), recessive fitness (*h* = 0), and the *h*(*s*) = 0.5/(1–7071.07*s*) relationship [14] separately, using the same DFE for *s* for each simulation. All simulations were scaled by a factor of *c* = 5.

### Simulations of Arabidopsis genomic structure

In simulations of fixed chromosome structure from the genome of *Arabidopsis thaliana*, we fixed the structure to 29.1 Mb from chromosome 1 (chr1:488,426–29,588,426). The exon ranges were defined using the Araport11 annotations [85] and the recombination map using Salomé et al. [86]. The per base pair mutation rate was constant and set to *μ* = 7×10^−9^ and we again set nonsynonymous and synonymous mutations to occur at a ratio of 2.31:1. The selection coefficients (*s*) of new nonsynonymous mutations were drawn from the gamma distribution estimated by Huber et al. [31] (shape parameter 0.185 and E[*s*] = -0.00048655). We simulated dominance with the *h*(*s*) relationship estimated by that study: *h*(*s*) = 1((1/0.987)– 39547*s*). Simulations were scaled at *c* = 100, but we note that we could not test the difference between *c* = 100 and smaller scaling factors (e.g. *c* = 50) due to limits on computational time.

### Avoiding heterosis in the additive fitness model

Computing fitness as additive (*h* = 0.5) within a locus but multiplicative across loci was problematic for our simulations because it created heterosis in admixed individuals. This occurred because the product of a fitness decrease reduces fitness less than the sum of a fitness decrease. As a simple example, imagine two additive deleterious alleles are in a single individual, each with selection coefficient *s* where *s* is the absolute value of the selection coefficient. If they are found as a single homozygous site, the fitness decrease is usually computed as 1-*s*. If they are found as two heterozygous sites, the fitness would be computed as (1–0.5*s*)^2^ = 1-*s*+0.25*s*^2^. The fitness of the heterozygous individual is larger than the homozygous individual by 0.25*s*^2^, despite carrying the same number of deleterious variants. Because admixed individuals are more likely to carry deleterious alleles as heterozygotes than non-admixed individuals, the fitness of the admixed individuals is always higher than a non-admixed individual in the above computation of fitness, even when the number of deleterious variants per individual is the same.

Our intent was to examine the contribution of deleterious variation to selection on introgressed ancestry, but we have identified an inherent advantage of heterozygosity in the additive model that biased the direction of selection to favor introgressed ancestry. To address this, we computed heterozygote fitness at a locus as 1-*hs* and homozygote fitness as (1–0.5*s*)^2^, and the fitnesses across loci were computed multiplicatively. In the additive case (*h* = 0.5), an individual’s fitness was then multiplicative across all deleterious variants, such that an individual *j* carrying *i* variants each with selection coefficient *s*_*i*_ had fitness *w*_*i*_:

$$w_{j}\;=\;\prod_{i}\left(1+{0.5s}_{i}\right)$$

Fitness was then monotonically related to the number of deleterious variants regardless of zygosity while remaining approximately equivalent to additive fitness. This computation in essence created a slight underdominance-like effect, but importantly this effect was caused by the difference in homozygous fitness rather than a difference in heterozygote fitness (*i*.*e*. the dominance coefficient). In practice, the difference in homozygous fitness is negligible for weakly deleterious alleles and strongly deleterious alleles are unlikely to be found as homozygotes. Therefore, the overall underdominance effect should be minimal. To confirm this, we simulated 100Mb of human chromosome 1 in an equilibrium population, with selection coefficients drawn from a gamma DFE with the two fitness models. The frequency spectrum was unaffected by our calculation of fitness (S9 Fig), suggesting our simulations approximate the standard additive model well.

We used the same calculation for additive and partially recessive fitness models for consistency when simulating the *h*(*s*) relationship. Completely recessive fitness (*h* = 0) was computed the standard way, that is, as 1-*s*_*i*_ when homozygous for the deleterious allele and as 1 otherwise.

### Selection on the X chromosome

We modeled fitness of the sex chromosomes following the framework described by Charlesworth et al. [53] and Veeramah et al. [52], with a slight modification to preserve the multiplicative fitness scenario described for the autosome. The specific fitness models for each dominance scenario—additive, recessive, and with the *h*(*s*) function—are presented in S2 Table. Importantly, the fitness of females that are homozygous and males that have the selected allele are the same, and, in the additive model, heterozygous females have an intermediate fitness. This models dosage compensation in females, assuming levels of gene expression map to the same fitness values for males and females.

## Supporting information

S1 Fig The change in the mean fitness of the donor and recipient subpopulation in each model. The mean (solid line) is shown for 200 simulation replicates. The vertical grey line depicts the time of gene flow. Different colors denote distinct recombination rates used in the simulations. The left two panels depict simulations with recessive mutations (*h* = 0) while the right two panels show simulations with additive mutations (*h* = 0.5). Variants that are fixed in both subpopulations are not considered in the calculation of fitness. The model numbers refer to the models shown in Fig 1.
(TIF)

S2 Fig The change in the mean number of derived deleterious sites (*s*<0) per haplotype in each model in the recipient subpopulation. The mean (solid line) is shown for 200 simulation replicates. The vertical gray line depicts the time of gene flow. Different colors denote distinct recombination rates used in the simulations. The left panel shows simulations with recessive mutations (*h* = 0) while the right panel shows simulations with additive mutations (*h* = 0.5). Variants that are fixed in both subpopulations are not counted. The model numbers refer to the models shown in Fig 1.
(TIF)

S3 Fig The change in the mean number of homozygous derived deleterious sites per individual in the recipient subpopulation. The mean (solid line) is shown for 200 simulation replicates. The vertical gray line depicts the time of gene flow. Different colors denote distinct recombination rates used in the simulations. The left panel shows simulations with recessive mutations (*h* = 0) while the right panel shows simulations with additive mutations (*h* = 0.5). Variants that are fixed in both subpopulations are not counted. The model numbers refer to the models shown in Fig 1.
(TIF)

S4 Fig The relationship of the split time to measures of divergence between subpopulations in models 0 and 4. The vertical gray lines represent the time between population divergence and admixture (100, 250, 500, 1,000, 2,500, 5,000, 10,000, 20,000, 25,000, 30,000, 35,000, and 40,000 generations) in the demographic models as depicted in Fig 4. Model numbers refer to Fig 1. (A) Population split time and population size impact the number of variants private to each subpopulation at the time of admixture. The numbers of variants that are private to the donor and recipient subpopulations, or shared between subpopulations, are shown for 200 simulation replicates and two demographic models. (B) *F*_*ST*_ increases continuously in Models 0 and 4 after the split. Increased drift in Model 4 drives larger increases in *F*_*ST*_.
(TIF)

S5 Fig The genomic landscape of introgression in one simulation replicate with human genomic structure. The frequency of ancestry that is introgression-derived is shown for non-overlapping 100 kb windows in a simulated 100 Mb region of chromosome 1. The model numbers refer to the models shown in Fig 1. Points represent a single value for each 100 kb window and lines are loess curves fitted to the data. The horizontal dashed black dashed line represents the initial frequency of introgression-derived ancestry, *p*_*I*_ = 0.05. Vertical blue bars represent genes in which deleterious mutations can occur. Red curves denote the results for recessive mutations, orange curves show the results for additive mutations, and blue curves show the results for simulations with a *h*(*s*) relationship.
(TIF)

S6 Fig The density distribution of introgression desert lengths for simulations of human chromosome 1. Introgression deserts are segments without any hybrid ancestry. Model numbers refer to the models shown in Fig 1.
(TIF)

S7 Fig The genomic landscape of introgression in one simulation replicate with *Arabidopsis* genomic structure. The frequency of ancestry that is introgression-derived is shown for non-overlapping 100 kb windows in a simulated 100 Mb region of chromosome 1. The model numbers refer to the models shown in Fig 1. Points represent a single value for each 100 kb window and lines are loess curves fitted to the data. The horizontal dashed black line represents the initial frequency of introgression-derived ancestry, *p*_*I*_ = 0.05. Vertical blue bars represent genes in which deleterious mutations can occur. Blue curves show the results for simulations with a *h*(*s*) relationship.
(TIF)

S8 Fig Scaled simulations accurately reproduce introgression in simulations with no scaling. The average frequency of introgressed ancestry (*p*_*I*_) of 100 simulation replicates of Model 4 and additive fitness (*h* = 0.5) is plotted through time. The average *p*_*I*_ for four different scaling factors (*c* = 1, 2, 5, and 10) is shown. The simulations in this study use *c* = 5 unless mentioned otherwise. Details are provided in the **Methods**.
(TIF)

S9 Fig The site frequency spectrum of nonsynonymous variants is the same for additive and multiplicative fitness models. The site frequency spectrum (SFS) is the same when fitness is calculated as multiplicative within a locus as it is when fitness is additive within a locus. Simulations were of an equilibrium population with 100 Mb of human genomic structure in a sample of size *n* = 2,000 chromosomes. Confidence intervals represent standard errors computed from 100 simulation replicates. All variants at frequency ≥25 are summed together in the last entry of the SFS.
(TIF)

S1 Table Demographic parameters of the simulated models shown in Fig 1. (DOCX)

S2 Table Fitness model for sex chromosomes. (DOCX)
