# Supplementary figures and images for: Deleterious variation shapes the genomic landscape of introgression
Source: PLoS Genet. 2018 Oct 22;14(10):e1007741. doi: 10.1371/journal.pgen.1007741 (PMC6233928; doi:10.1371/journal.pgen.1007741)

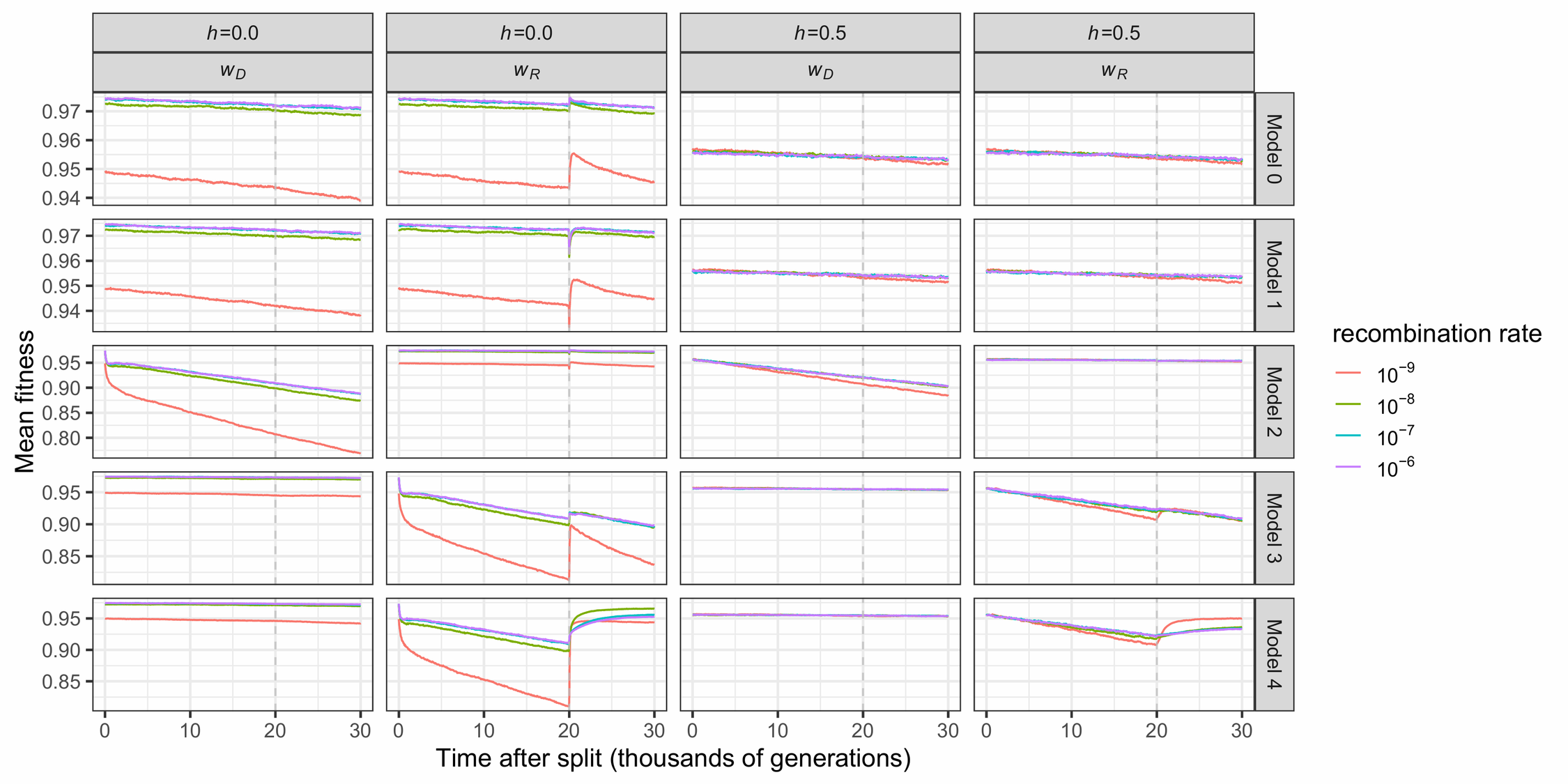

Supplement: S1 Fig — The mean (solid line) is shown for 200 simulation replicates. The vertical grey line depicts the time of gene flow. Different colors denote distinct recombination rates used in the simulations. The left two panels depict simulations with recessive mutations (h = 0) while the right two panels show simulations with additive mutations (h = 0.5). Variants that are fixed in both subpopulations are not considered in the calculation of fitness. The model numbers refer to the models shown in Fig 1. (TIF) [file pgen.1007741.s001.tif]

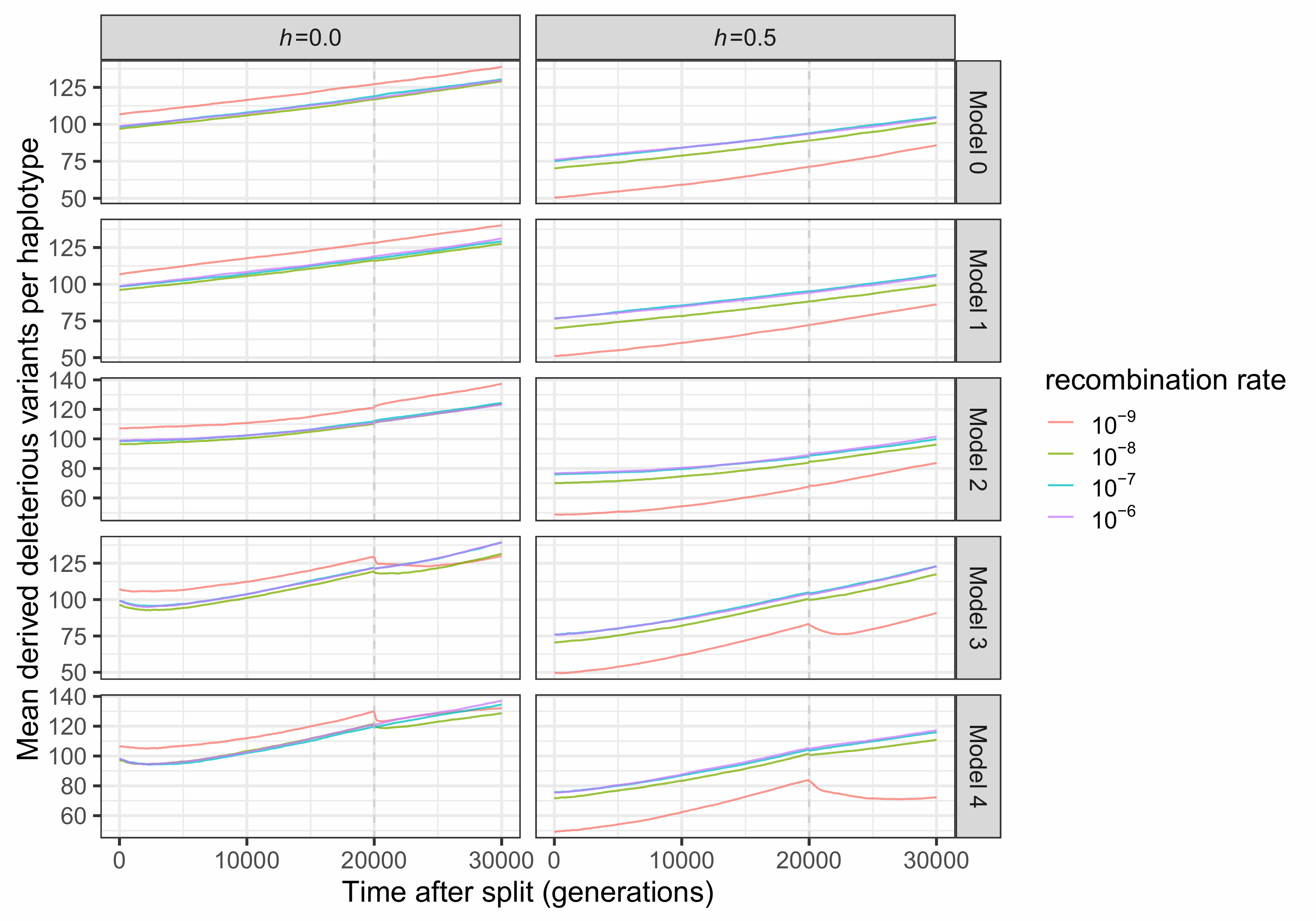

Supplement: S2 Fig — The mean (solid line) is shown for 200 simulation replicates. The vertical gray line depicts the time of gene flow. Different colors denote distinct recombination rates used in the simulations. The left panel shows simulations with recessive mutations (h = 0) while the right panel shows simulations with additive mutations (h = 0.5). Variants that are fixed in both subpopulations are not counted. The model numbers refer to the models shown in Fig 1. (TIF) [file pgen.1007741.s002.tif]

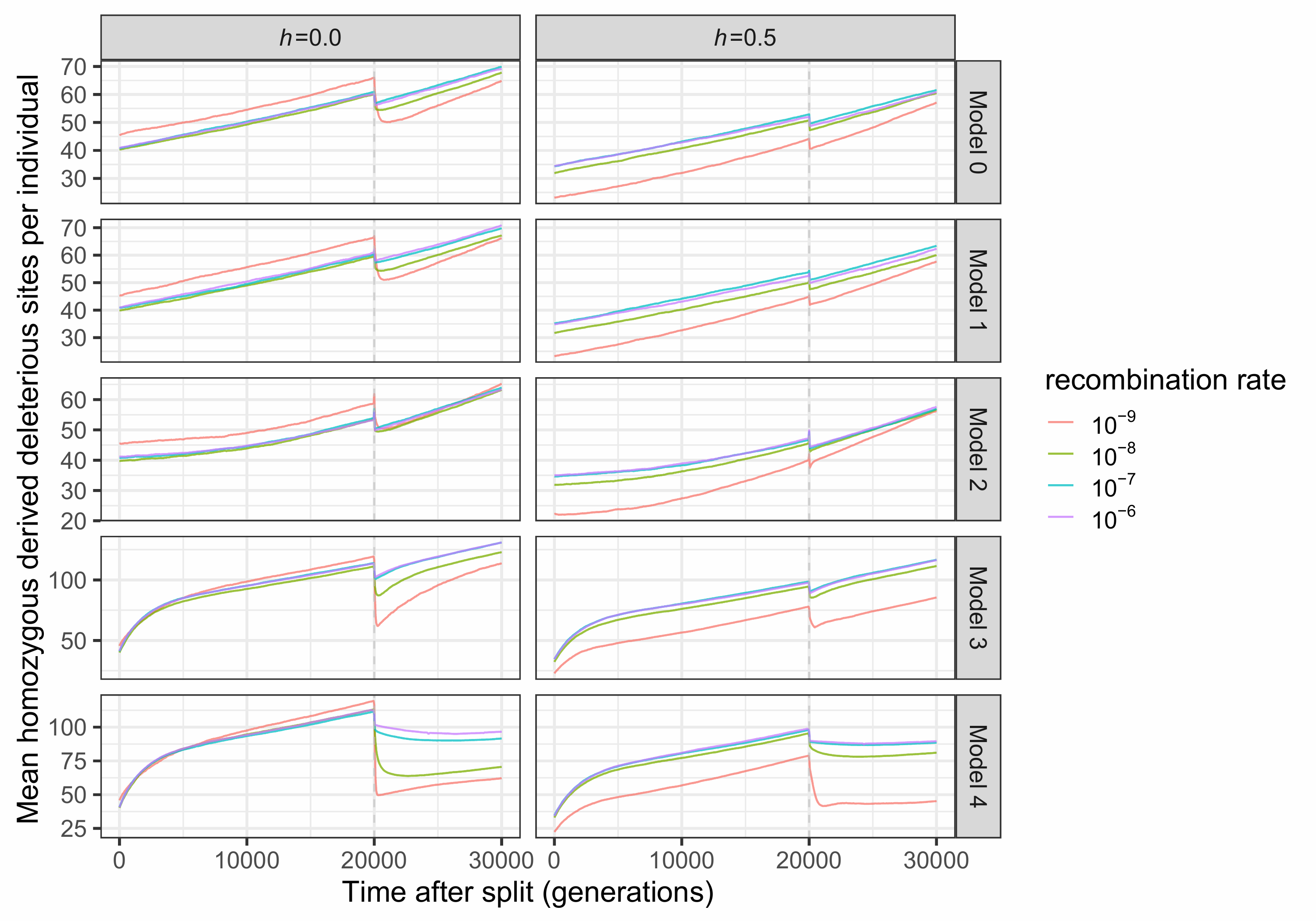

Supplement: S3 Fig — The mean (solid line) is shown for 200 simulation replicates. The vertical gray line depicts the time of gene flow. Different colors denote distinct recombination rates used in the simulations. The left panel shows simulations with recessive mutations (h = 0) while the right panel shows simulations with additive mutations (h = 0.5). Variants that are fixed in both subpopulations are not counted. The model numbers refer to the models shown in Fig 1. (TIF) [file pgen.1007741.s003.tif]

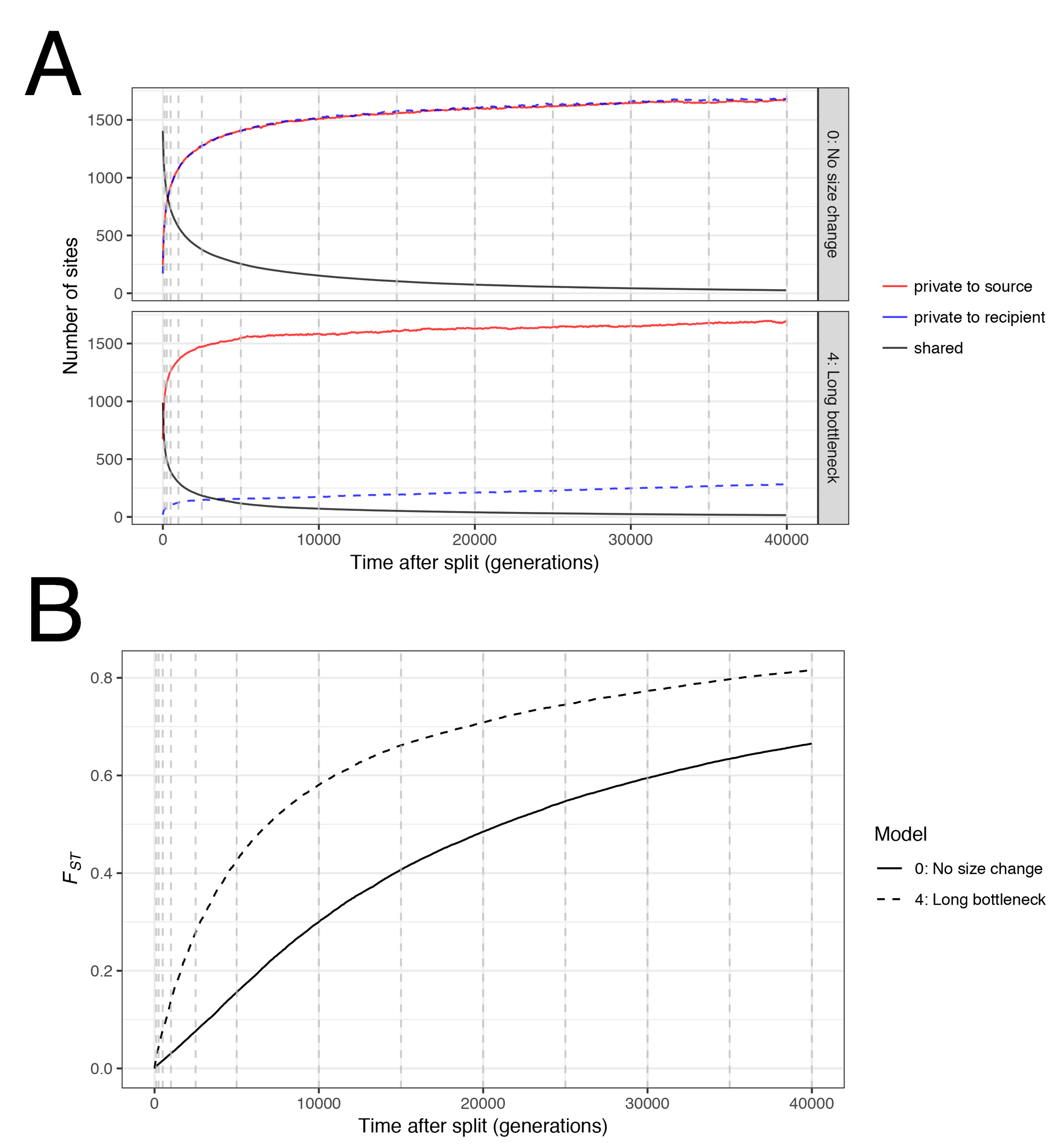

Supplement: S4 Fig — The vertical gray lines represent the time between population divergence and admixture (100, 250, 500, 1,000, 2,500, 5,000, 10,000, 20,000, 25,000, 30,000, 35,000, and 40,000 generations) in the demographic models as depicted in Fig 4. Model numbers refer to Fig 1. (A) Population split time and population size impact the number of variants private to each subpopulation at the time of admixture. The numbers of variants that are private to the donor and recipient subpopulations, or shared between subpopulations, are shown for 200 simulation replicates and two demographic models. (B) FST increases continuously in Models 0 and 4 after the split. Increased drift in Model 4 drives larger increases in FST. (TIF) [file pgen.1007741.s004.tif]

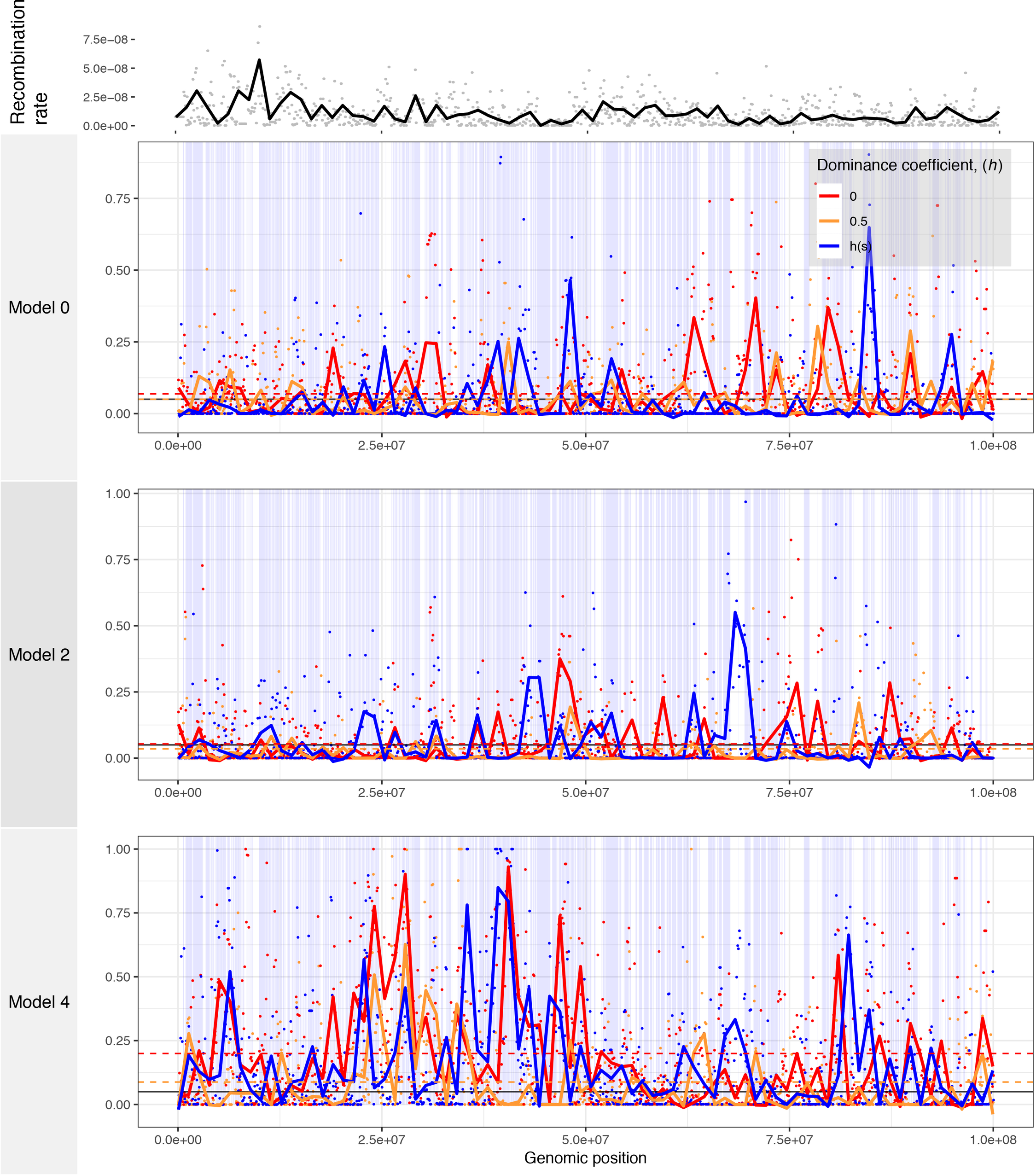

Supplement: S5 Fig — The frequency of ancestry that is introgression-derived is shown for non-overlapping 100 kb windows in a simulated 100 Mb region of chromosome 1. The model numbers refer to the models shown in Fig 1. Points represent a single value for each 100 kb window and lines are loess curves fitted to the data. The horizontal dashed black dashed line represents the initial frequency of introgression-derived ancestry, pI = 0.05. Vertical blue bars represent genes in which deleterious mutations can occur. Red curves denote the results for recessive mutations, orange curves show the results for additive mutations, and blue curves show the results for simulations with a h(s) relationship. (TIF) [file pgen.1007741.s005.tif]

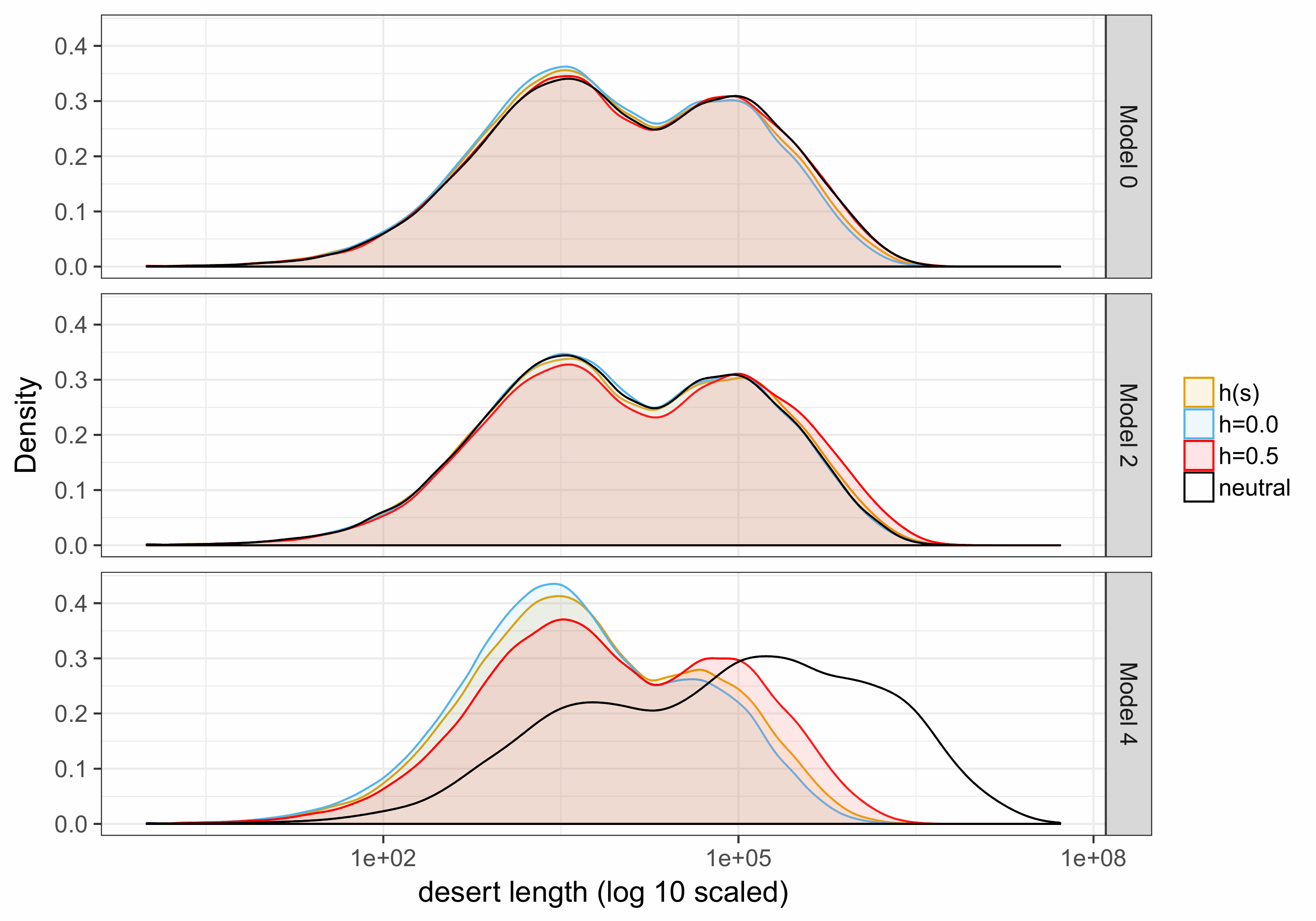

Supplement: S6 Fig — Introgression deserts are segments without any hybrid ancestry. Model numbers refer to the models shown in Fig 1. (TIF) [file pgen.1007741.s006.tif]

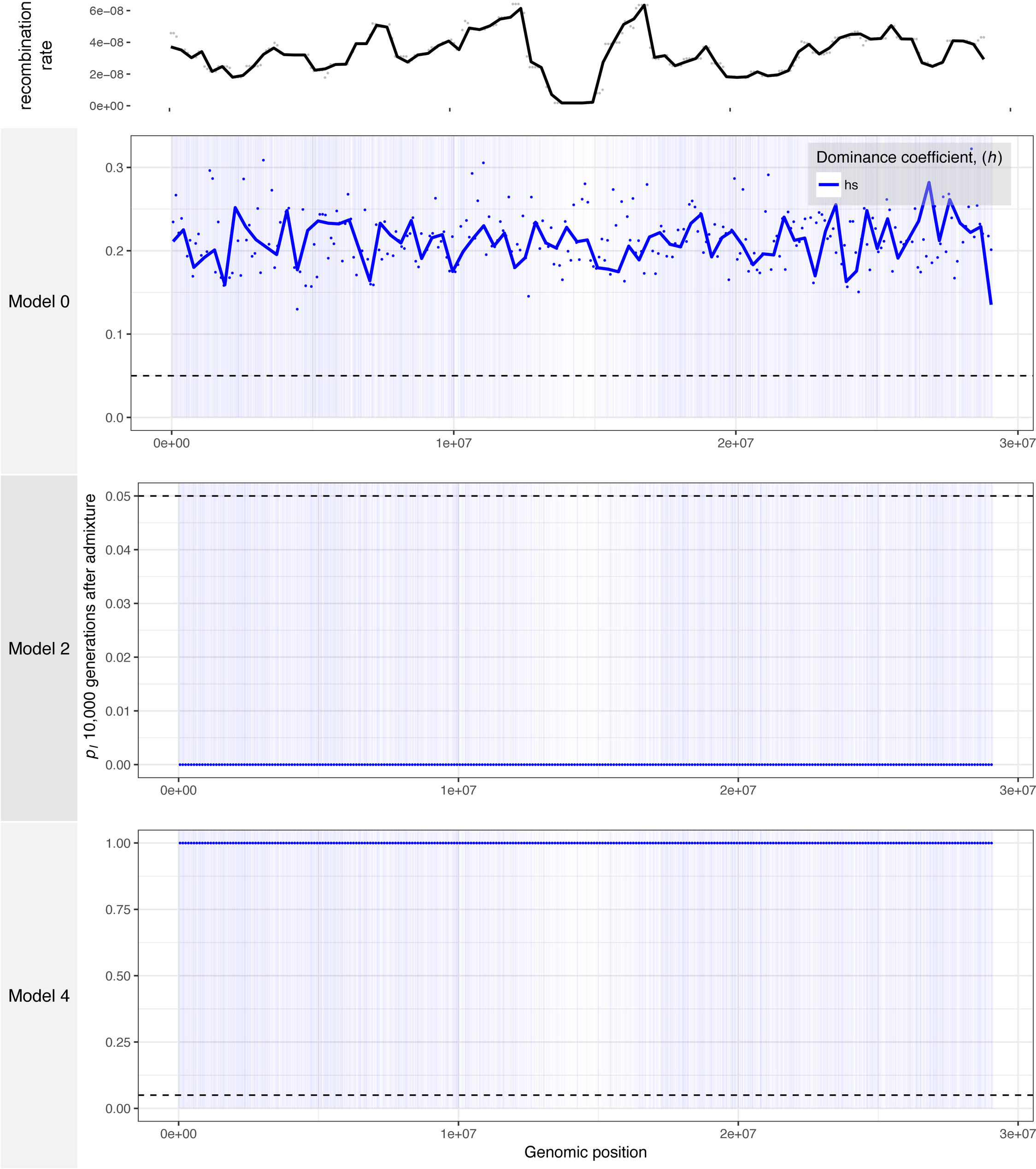

Supplement: S7 Fig — The frequency of ancestry that is introgression-derived is shown for non-overlapping 100 kb windows in a simulated 100 Mb region of chromosome 1. The model numbers refer to the models shown in Fig 1. Points represent a single value for each 100 kb window and lines are loess curves fitted to the data. The horizontal dashed black line represents the initial frequency of introgression-derived ancestry, pI = 0.05. Vertical blue bars represent genes in which deleterious mutations can occur. Blue curves show the results for simulations with a h(s) relationship. (TIF) [file pgen.1007741.s007.tif]

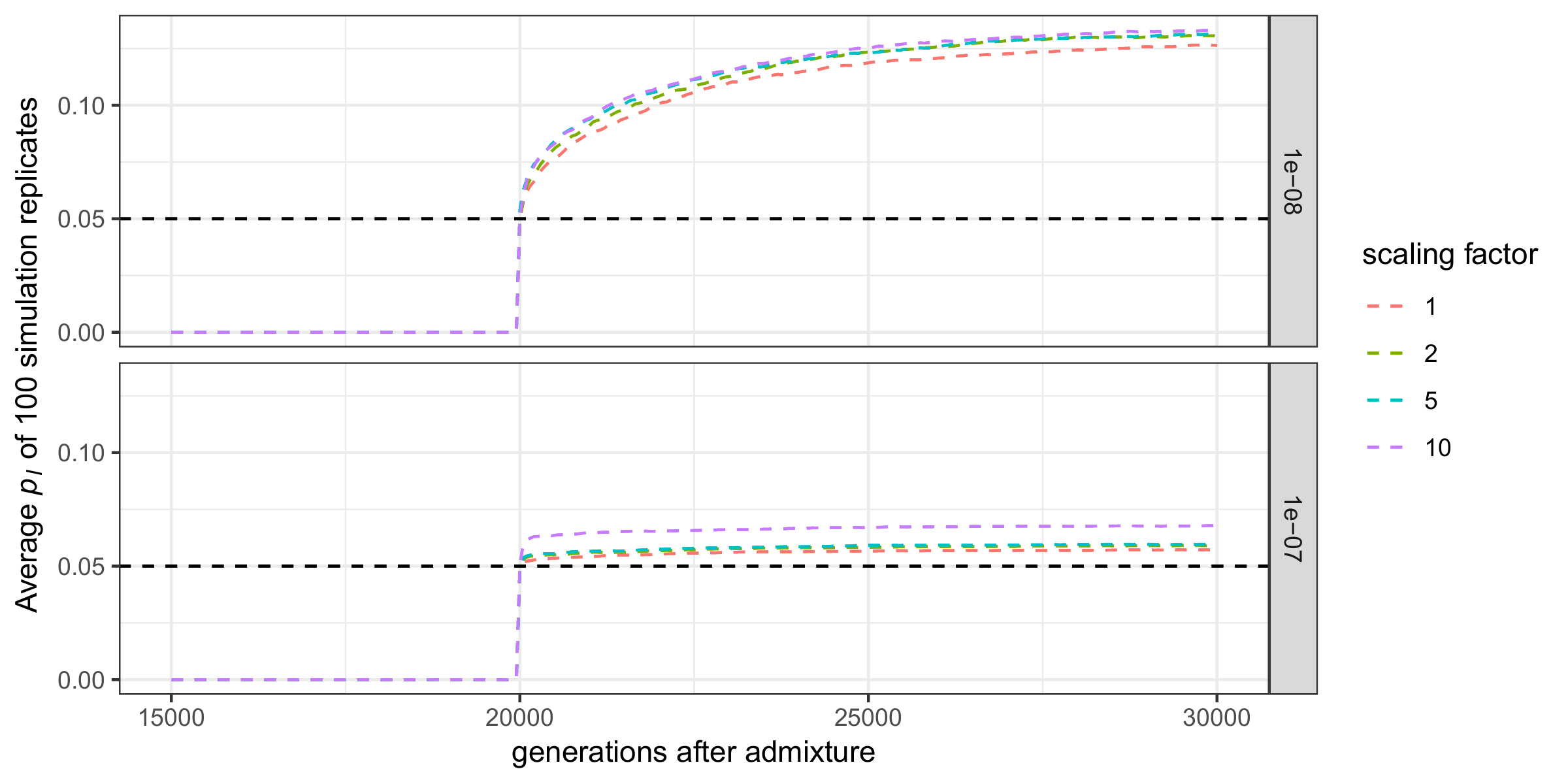

Supplement: S8 Fig — The average frequency of introgressed ancestry (pI) of 100 simulation replicates of Model 4 and additive fitness (h = 0.5) is plotted through time. The average pI for four different scaling factors (c = 1, 2, 5, and 10) is shown. The simulations in this study use c = 5 unless mentioned otherwise. Details are provided in the Methods. (TIF) [file pgen.1007741.s008.tif]

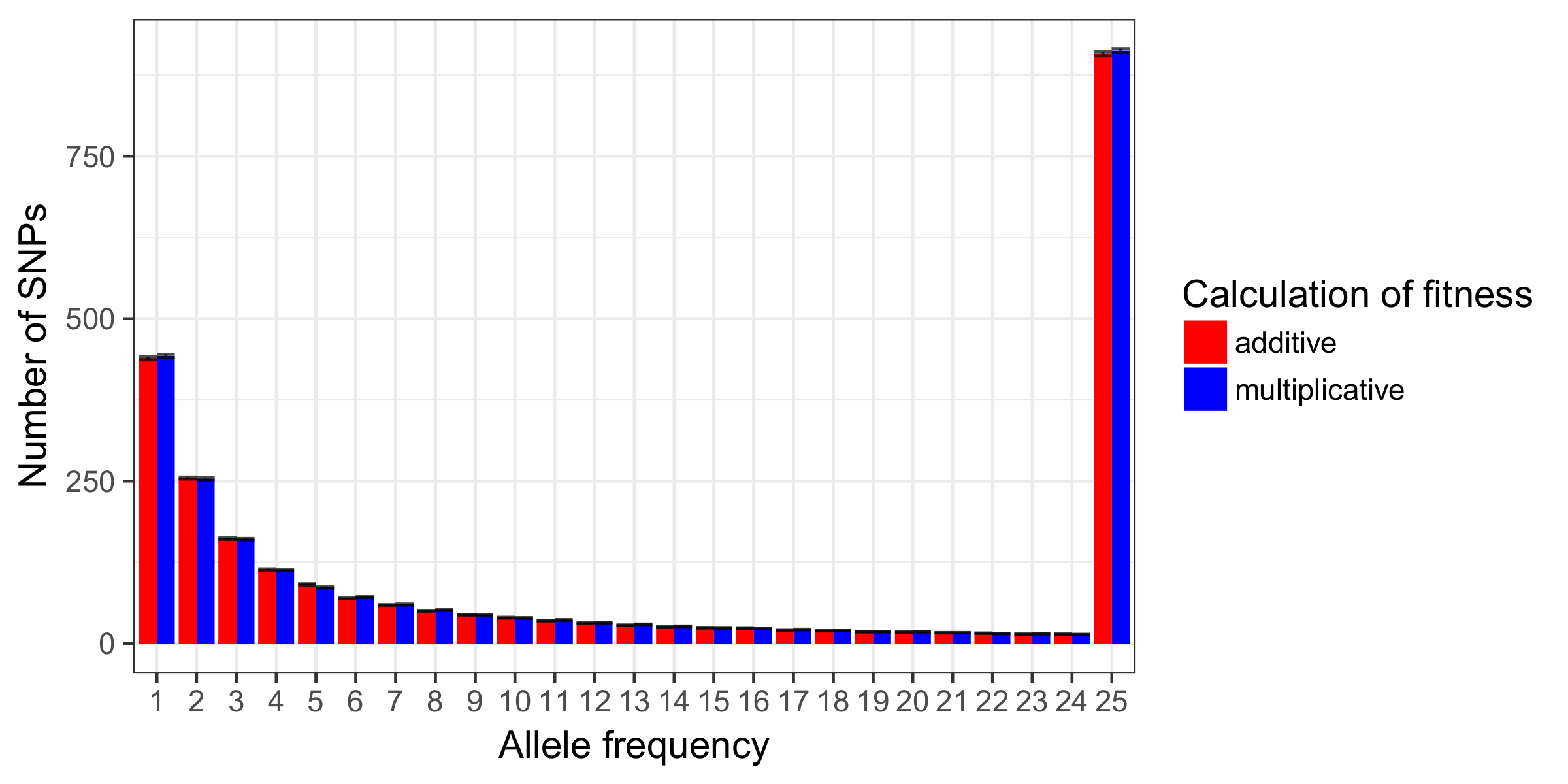

Supplement: S9 Fig — The site frequency spectrum (SFS) is the same when fitness is calculated as multiplicative within a locus as it is when fitness is additive within a locus. Simulations were of an equilibrium population with 100 Mb of human genomic structure in a sample of size n = 2,000 chromosomes. Confidence intervals represent standard errors computed from 100 simulation replicates. All variants at frequency ≥25 are summed together in the last entry of the SFS. (TIF) [file pgen.1007741.s009.tif]
