# Supplementary material for: Deleterious variation shapes the genomic landscape of introgression
Source: PLoS Genet. 2018 Oct 22;14(10):e1007741. doi: 10.1371/journal.pgen.1007741 (PMC6233928; doi:10.1371/journal.pgen.1007741)
Supplement: S1 Table — (DOCX) [file pgen.1007741.s010.docx]

**Table S1. Demographic parameters of the simulated models shown in Figure 1.**

| **Model** | *N_S_* | *N_RB_* | *N_R_* | *T_m_* | *T_RB_* | *T_split_* |
| --- | --- | --- | --- | --- | --- | --- |
| Model 0 | 1 | -- | 1 | 0.5 | -- | 1.5 |
| Model 1 | 1 | 0.1 | 1 | 0.5 | 0.5025 | 1.5 |
| Model 2 | 0.1 | -- | 1 | 0.5 | -- | 1.5 |
| Model 3 | 1 | -- | 0.1 | 0.5 | -- | 1.5 |
| Model 4 | 1 | 0.1 | 1 | 0.5 | -- | 1.5 |

NOTE.—All population sizes are relative to the ancestral population size (*N_A_*), where *N_A_*=10,000 diploids unless specified otherwise. All times are in units of generations/(2*N_A_*) from the present day. Parameters are defined as follows. *N_S_*: size of the source subpopulation, *N_RB_*: size of the bottleneck in the recipient population, *N_R_*: size of the recipient population, *T_m_*: time of migration, *T_RB_*: time at which the bottleneck in the recipient population began, *T_split_*: time at which the subpopulations diverged
