# Supplementary material for: Deleterious variation shapes the genomic landscape of introgression
Source: PLoS Genet. 2018 Oct 22;14(10):e1007741. doi: 10.1371/journal.pgen.1007741 (PMC6233928; doi:10.1371/journal.pgen.1007741)
Supplement: S2 Table — (DOCX) [file pgen.1007741.s011.docx]

**Table S2. Fitness model for sex chromosomes.**

|  | **genotype** | | |
| --- | --- | --- | --- |
| **dominance** | XX heterozygote | XX homozygote | XY |
| Additive (*h=*0.5) | (1+*hs*) | (1+*hs*)^2^ | (1+*hs*)^2^ |
| Recessive | 1 | (1+0.5*s*)^2^ | (1+0.5*s*)^2^ |
| *h*(*s*) | (1+*hs*) | (1+0.5*s*)^2^ | (1+0.5*s*)^2^ |

NOTE.*—h* denotes the dominance coefficient and (1+0.5*s*)^2^ is approximately equal to (1-*s*).
